# Goals in Nutrition Science 2025–2030

**DOI:** 10.3389/fnut.2026.1784021

**Published:** 2026-03-09

**Authors:** Elliot M. Berry, Barbara R. Cardoso, Sean B. Cash, Alejandro Cifuentes, Maria Carmen Collado, Johannes le Coutre, J. Bruce German, Elena Ibáñez, Mark Lawrence, David C. Nieman, Igor Pravst, David Raubenheimer, Michael Rychlik, Andrew Scholey, Annalisa Terranegra, Angela M. Zivkovic

**Affiliations:** 1 Faculty of Medicine, Braun School of Public Health, Hebrew University-Hadassah Medical School, Jerusalem, Israel; 2 Department of Nutrition, Dietetics and Food, Victorian Heart Institute, Monash University, Notting Hill, VIC, Australia; 3 Friedman School of Nutrition Science and Policy, Tufts University, Boston, MA, United States; 4 Foodomics Laboratory, Institute of Food Science Research, CIAL-CSIC, Madrid, Spain; 5 Institute of Agrochemistry and Food Technology-National Research Council (IATA-CSIC), Valencia, Spain; 6 School of Chemical Engineering and Australian Human Rights Institute, University of New South Wales (UNSW), Sydney, NSW, Australia; 7 Department of Food Science and Technology, University of California, Davis, Davis, CA, United States; 8 School of Exercise and Nutrition Sciences, Institute for Physical Activity and Nutrition (IPAN), Deakin University, Geelong, VIC, Australia; 9 Human Performance Laboratory, Department of Biology, Appalachian State University, North Carolina Research Campus, Boone, NC, United States; 10 Nutrition and Public Health Research Group, Institute of Nutrition, Ljubljana, Slovenija; 11 School of Biological Sciences and Charles Perkins Centre, The University of Sydney, Sydney, NSW, Australia; 12 Technical University of Munich (TUM), Freising, Germany; 13 Northumbria University, Newcastle-upon-Tyne, United Kingdom; 14 Translational Medicine Department, Sidra Medicine, Doha, Qatar; 15 Department of Nutrition, University of California, Davis, Davis, CA, United States

**Keywords:** food security, food system, health, nutrition science, Sustainable Development Goals (SDGs), sustainability

## Abstract

Already in its third edition, the Goals in Nutrition Science platform covers a five-year timeframe per volume, thus spanning 15 years from 2015 to 2030 (1, 2). This period aligns with the Sustainable Development Goals, and, in practice, these 5-year updates do capture major shifts in the field. As the second quarter of the 21st century unfolds, it increasingly appears that much of the widely promoted food technology has not delivered or is not yet ready. Nutrition, food security, and sustainability are therefore best treated as inseparable challenges within complex, adaptive food systems, where progress depends on addressing biology, behavior, markets, policy, and environmental constraints together rather than through isolated, linear interventions. Nutrition science matters because it sits at the hinge between human biology and the real-world conditions that determine what people can access, afford, choose, and safely consume. As food systems become more interconnected and more exposed to climate, conflict, and market volatility, the field is shifting from mainly reductionist problem solving toward approaches that can handle feedback, tradeoffs, and equity in context. Pursuing the goals set out here is not only a scientific agenda, but a planetary health imperative: sustainable food systems must secure current and future nutrition while balancing environmental stewardship, health, and socio-economic stability across the pathway from production to consumption and waste. Overall, the agenda points toward a new chapter of nutrition science that integrates the right level of complexity by combining deep disciplinary insight with better integrated systems approaches, and by mobilizing coordinated action.

Johannes le Coutre, Field Chief Editor, Frontiers in Nutrition.

The contributions in this article converge on a shared premise: nutrition, food security, and sustainability are interconnected properties of complex, adaptive food systems shaped by shocks, incentives, governance, and environmental limits. Priority actions include strengthening resilience and food security, and embedding nutrition goals into trade, industrial, and regulatory policy as protectionism and supply chain volatility reshape diets, prices, and equity. Governance is the difference between promise and harm, requiring standards and fairness mechanisms that keep pace with innovation.

Diet diversification is a major opportunity, but it must protect quality, cultural integrity, and fair benefit sharing as plant-based foods and foods of Indigenous origin scale. The agenda moves beyond single nutrient debates toward whole diet and system effects, with ultra processed foods a continuing fault line. Emerging contaminants, especially micro and nanoplastics, should be integrated into food safety management and addressed through coordinated action across processing, materials, and packaging reform aligned with upstream environmental measures.

Discovery and translation are increasingly data enabled. AI supported pattern recognition and context specific dietary guidance depend on comprehensive, high resolution food composition data as foundational infrastructure. Precision nutrition can move beyond one-size-fits-all advice when recommendations are mechanistically grounded, outcome validated, and feasible at scale within real world constraints, including access, affordability, culture, and policy. Microbiome science advances through standardized methods and validated biomarkers linked to clinically meaningful endpoints, supporting a shift from association toward causality.

Evidence gaps remain for performance nutrition and for brain health priorities, including long COVID, where stronger interdisciplinary programs and better trials are needed to connect nutrition with cognition, mood, fatigue, and functional outcomes. The rapid expansion of GLP 1 receptor agonists has implications for eating behaviors, food choice, and potential micronutrient risks, underscoring the need to integrate pharmacotherapy trends into nutrition research, guidance, and monitoring. *One Health* provides a unifying frame linking human, animal, and ecosystem health, clarifying why nutrition goals must align with food safety, environmental stewardship, and sustainable production. Interdisciplinary systems thinking is the practical method for coordinating action across civil society, science, industry, and policy to deliver durable population benefit within planetary limits.

## Linking food security and sustainable food systems as *complex adaptive systems*

### (Elliot M. Berry)

Food security ensures that all people at all times have consistent, equitable access to sufficient, safe, affordable and nutritious food for a healthy, active life, in harmony with planetary boundaries (3, 4). This concept now embraces six dimensions: availability, accessibility, utilization, stability, sustainability, and agency—community involvement—reflecting how food security spans from individual nutrition to global systems (5). Achieving worldwide food security is directly linked to the evolution of sustainable food systems (6).

#### Food systems as Complex Adaptive Systems (CAS)

**Figure 1:**
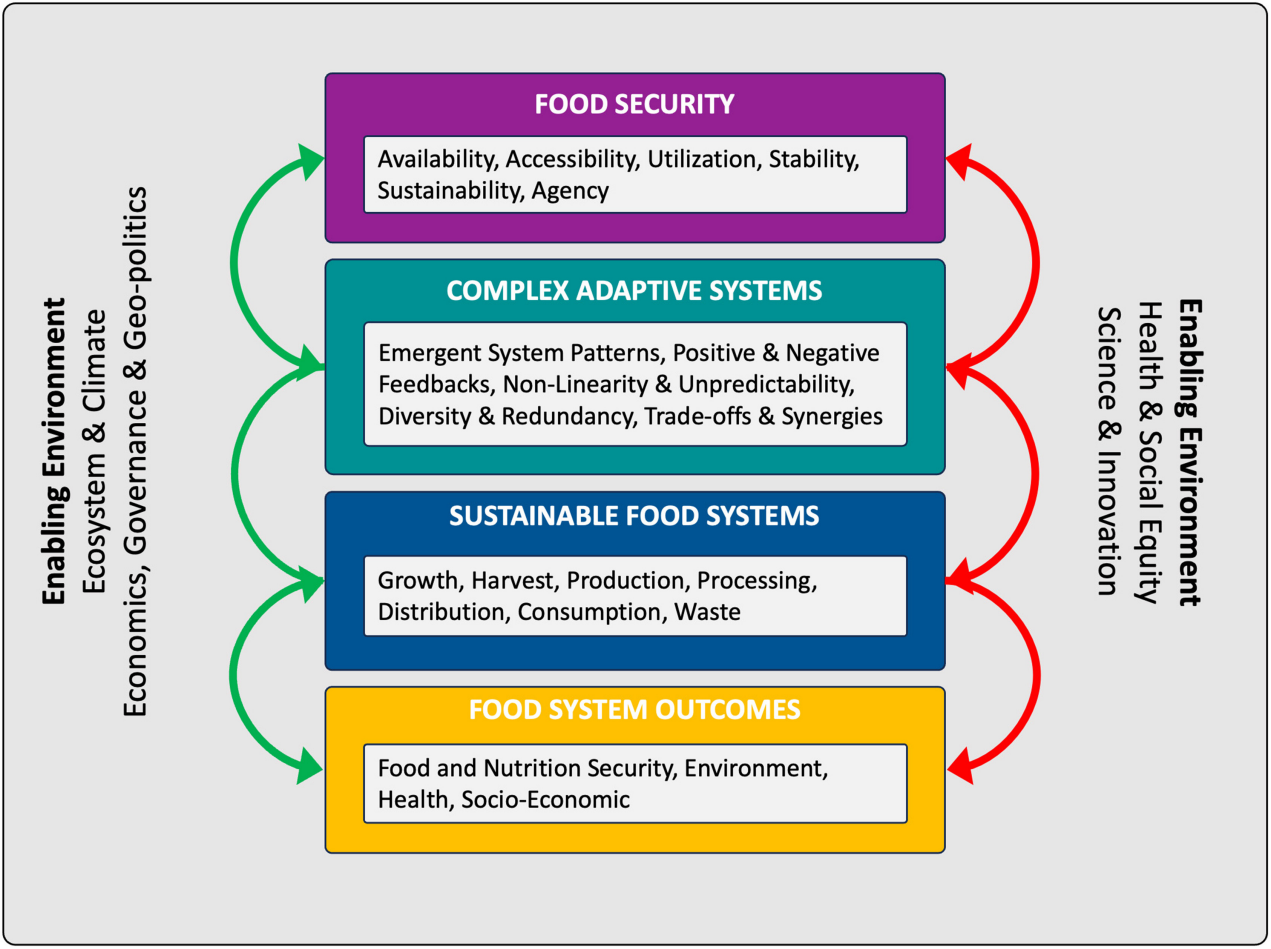
Food security, with its six dimensions, and Sustainable Food Systems and their outcomes, are inter-related as dynamic Complex Adaptive Systems (CAS). The enabling environments are themselves CAS.

Addressing food insecurity exemplifies the difficulty of “wicked problems”: these challenges are multidimensional, context-dependent, and marked by conflicting stakeholder interests, making simple solutions elusive (7, 8). Climate change, poverty, political instability, and global supply chain disruptions underscore the intricate, unpredictable nature of food system vulnerabilities. To address such problems, policymakers and practitioners are turning increasingly toward Complex Adaptive Systems (CAS) theory (see Figure 1), which reveals the non-linear, emergent dynamics that shape outcomes at every level (9, 10). A sustainable food system secures both current and future nutrition, covering the entire pathway from primary production to consumption and waste, and must balance nutrition security, environmental stewardship, health, and socio-economic stability (11).

Within the CAS framework, food systems comprise diverse agents—farmers, processors, retailers, regulators, and consumers—interacting dynamically in local, regional, and global contexts. These networks are characterized by interconnected subsystems, positive and negative feedback mechanisms, and adaptive behaviors, producing unpredictable outcomes that are not apparent from analysis of individual components (12–14).

For example, a drought in one region can trigger local crop failures, ripple through markets, cause price spikes, and even affect access to food far away due to the integrated nature of global supply chains. The recent conflicts in Ukraine and the Middle East wars, with disruptions to major shipping corridors, including the Suez Canal, have damaged food supply chains and increased prices world-wide.

#### Policy interventions

Technological advances, expanded trade, and increasingly complex supply chains have boosted productivity but also introduced new vulnerabilities and dependencies (15, 16). As a result, policy and governance must address not only efficiency and equity but systemic risk, adaptability, and resilience (17).

Policy interventions should facilitate diversity and redundancy within food systems. By promoting crop and market variety, decentralizing supply chains, and strengthening community-based models such as farmers' markets and local cooperatives, food systems become more robust against disruptions and shocks (18, 19). Adaptive governance is essential, requiring participatory approaches that engage stakeholders—farmers, civil society, private sector, and local authorities—in decision-making and responses to crisis and change (20, 21). Innovation by subtraction is also critical: simplifying supply chains, re-localizing processing, and prioritizing agroecological and low-input methods helps to avoid over-complexification with its associated risks (19, 22).

From a complex adaptive systems (CAS) perspective, innovation by subtraction enhances food-system resilience by reducing structural fragility rather than increasing functional capacity. This means strengthening food systems by removing unnecessary intermediaries and fragile global dependencies, creating simpler, more local, and more resilient networks.

In developed countries, subtraction focuses on shortening the distance between producers and consumers to reduce exposure to global supply shocks and inflation. Examples include social supermarkets and buyers' clubs in the USA and Europe, alternative food networks in France and the Czech Republic, and short supply chains that cut food miles while strengthening direct producer–consumer relationships.

In developing regions, subtraction targets costly imported inputs and high-loss transport systems to secure local food consumption. Examples include Kenyan farmers using Black Soldier Fly larvae as locally produced protein feed instead of imported grain; Jordan's Agriculture Resilience Programme, which supports smallholders with simple, localized irrigation rather than vulnerable centralized infrastructures; community and backyard farming in Togo, reducing reliance on national distribution networks; and shelf-life extension technologies in Uganda, such as inhibiting ripening enzymes, that reduce dependence on expensive and unreliable cold chains.

The responsible deployment of digital technology can enhance transparency, responsiveness, and local empowerment, but should avoid adding unnecessary layers of complexity that increase vulnerability (23). Policy frameworks should be grounded in reflexive learning—incorporating regular review, real-time adaptation, and systems research to anticipate and manage emergent risks (24, 25). Prioritizing equity is vital, with attention to nutrition outcomes, wage fairness, and inclusion of marginalized and indigenous populations in policy design; targeted safety nets and nutrition education should ensure resources reach those most in need (26).

Governments and organizations must also maintain strategic reserves and diversified import/export partners to insulate against global supply failures, actively cultivating emergency logistics and food stocks at multiple scales. Regional and cross-sector partnerships—combining government, business, NGOs, and local communities—are essential for codesigned, sustainable solutions (20). These alliances should integrate disaster resilience and climate adaptation into food policy from the outset (27), while indigenous knowledge and local perspectives inform ongoing efforts to build sustainable food systems (28).

#### Polycrisis impacts and the way forward

Crises such as the COVID-19 pandemic (29) and recent geopolitical conflicts (30) have sharply highlighted vulnerabilities in centralized, complex global food networks, underlining the value of distributed, diverse, locally empowered systems for recovery and resilience (31, 32). These events demonstrate that systems with locally anchored supply chains and strong redundancy can adapt and recover much faster from shocks, offering practical evidence for key policy directions.

The transformation of food systems requires a fundamental shift in metrics and incentives, moving beyond narrow measures of yield and short-term efficiency to value long-term system health, resilience, social justice, and sustainability. Subsidies, investment, and regulation should reward practices that enhance adaptability and equity. Integrated system science and CAS modeling (network analysis, system dynamics, agent-based modeling) should underpin all risk assessment and intervention planning, ensuring that feedback from implementation continuously informs policy development (25, 33).

#### Toward resilient food security policy

Adopting a CAS perspective in food policy is essential for transforming food systems to succeed under unpredictable global conditions. Resilient food systems require embracing uncertainty at all levels, facilitating adaptive governance, and designing interventions to promote diversity, redundancy, local empowerment, and multi-stakeholder participation.

Key policy recommendations include:

- Strengthening diversity and redundancy at all system levels.
- Enhancing local governance coherence and empowering community actors.
- Reducing complexity via localization and simplification of supply chains.
- Harnessing digital tools judiciously, as enablers not complicators.
- Embedding reflexive learning and rapid policy adaptation.
- Promoting equity and targeting resources for the vulnerable.
- Building partnerships and transdisciplinary solutions for resilience.

Ultimately, food security must be approached as a dynamic systems challenge, where policy emphasizes uncertainty, adaptation, diversity, and collaborative governance at every level. The future of food security will depend on the ability to promote resilience, encourage local empowerment, and support innovation across diverse contexts. This paradigm shift—from reactive to proactive, centralized to distributed, and singular to integrative—will be vital to achieving the goals set out in the UN Sustainable Development Agenda (34–36), enabling food systems to sustain not only present populations but also future generations in an unpredictable world.

#### Looking to 2030—The future beyond the Sustainable Development Goals

Since their adoption in 2015, the Sustainable Development Goals (SDGs) have driven global action on poverty reduction, access to education, improved maternal and child health, clean energy, and climate resilience (37). There has been a 39% reduction in new HIV infections and malaria prevention efforts have saved 12.7 million lives. Over 110 million more children are in school, and the gender gap in education continues to shrink with more girls finishing school. Electricity access has reached 92% of the world's population while internet use has jumped from 40% in 2015 to 68% in 2024. Over half the population now benefits from some form of social protection and women's representation in parliament has increased to 27%. Conservation efforts have doubled protected ecosystem areas. Many countries have integrated the SDGs into national policies, fostering collaboration among governments, businesses, and civil society.

However, challenges remain—inequality persists, climate change impacts are escalating, and the COVID-19 pandemic reversed gains in health and education. In addition, the geopolitical upheavals in Ukraine and the Middle East have disrupted food supply chains, increasing prices globally. Progress has been uneven, with over 800 million people still living in extreme poverty. One in 11 people still face hunger and billions live without safe drinking water and sanitation. Climate records are being shattered, with 2024 the hottest year in history and CO_2_ levels are the highest in over two million years. Women continue to shoulder 2.5 times more unpaid care work than men, and persons with disabilities remain poorly helped. Over 120 million people are displaced—more than double the number in 2015. Debt service costs for low- and middle-income countries hit $1.4 trillion, draining resources from critical development. Only 35% of SDG targets are on track or making modest gains. Nearly half are progressing too slowly, and 18% are actually regressing. These numbers highlight the need for urgent action.

As the 2030 deadline approaches, attention is shifting toward accelerating implementation and shaping the “post-2030 agenda.” The United Nations emphasizes focusing on six areas: food systems, energy, digital transformation, education, jobs and social protection, and climate & biodiversity. Building on lessons from the SDGs, the next phase will aim for more accountability, financing innovation, and equitable progress that leaves no one behind. Stronger international cooperation and increased investment will be crucial for success beyond 2030 to ensure global food security, noting that “a food secure nation is a well-fed nation is a healthy nation is a productive, resilient and a sustainable nation” (38).

## Changing global markets for food: trade barriers and nutrition policy

### (Sean Cash and Igor Pravst)

The last few years have seen a reversal in the political support for globalization, with rises in both nationalist politics and trade protectionism in many countries (39). This trend toward “de-globalization” is generally not motivated by concerns around food or nutrition but nonetheless has major implications for both food security and dietary quality. Global food trade impacts human health by ensuring greater access to calories and satisfying unmet demand and increasing the availability of fresh fruits and vegetables, thereby enhancing food security, dietary diversity and nutrition; simultaneously, increased trade in minimally nutritious ultra-processed foods contributes to increasing rates of non-communicable diseases. While globalization historically supported food availability in all seasons, dietary diversification, and efficiency gains (40), it also introduced vulnerabilities related to supply chain disruptions, environmental externalities, and nutrition transitions toward ultra-processed foods.

The partial reversal and restructuring of global economic integration, reflected in higher tariffs and reduced cross-border trade, greater national sovereignty, and regionalization of supply chains, is increasingly evident in food systems and will impact food supply and nutrition worldwide. Such changes potentially limiting access to nutritious foods, challenge food security, and exacerbate health disparities. Furthermore, global shocks over the past decade—including the COVID-19 pandemic, geopolitical conflicts, and climate-related disruptions, have exposed the fragility of highly integrated food supply chains, leading to disruptions in production, transportation, and trade. These disruptions have consequently prompted protective national policies and reassessments of import dependence, demonstrating the vulnerability and complexity of global food systems in the face of external shocks (41, 42).

From a nutrition perspective, increasing trade barriers will alter food environments in ways that influence dietary patterns. Increasing tariffs increase the total price of food and lower the purchasing power of consumers (i.e., income effects) and their access to food, but also alter the relative prices of foods, influencing food choice (i.e., substitution effects) in ways that can have profound implications for dietary quality. These impacts can be felt in both relatively low-income and rich countries alike (43). For example, the current administration in the United States has been rapidly and inconsistently imposing new tariffs on its trading partners, engendering reciprocal tariffs in return. Much of what the United States imports from its neighbors is fresh fruits and vegetables (44), and concomitant restrictions on labor flows limit the ability of easily expanding domestic production. Limited access to imported fruits, vegetables, and protein sources can reduce diet quality, while higher prices disproportionately affect low-income populations. On the other hand, reduced inflows of ultra-processed foods into countries with less domestic food manufacturing may create opportunities for healthier dietary shifts, provided that local food systems are capable of adequately supporting such shifts.

#### Nutrition transition and dietary quality

Globalization has been an important driver of the nutrition transition, characterized by increased consumption of energy-dense, nutrient-poor foods and rising prevalence of obesity and diet-related non-communicable diseases (NCDs) (45, 46). De-globalization may slow or modify this transition by limiting the market penetration of multinational food corporations and processed food products. However, this will depend heavily on domestic food policies and industry responses. In the absence of strong nutrition-focused governance, de-globalization could also reinforce unhealthy dietary patterns if domestic food industries replicate globalized models of ultra-processing. Furthermore, given high usage of ultra processed foods, there is a risk that such products could be produced with even lower standards regarding the composition quality, particularly if production does not follow development processes, that also use nutrient profiling criteria. This interaction of regulation and dietary quality is an area of high research need. With increased de-globalization, chances for implementation globally (and regionally) harmonized nutrient profiling models could also be reduced—similarly affecting production incentives, food reformulation, and consumer information.

#### Implications for food labeling policies

While globalization might have presented an opportunity for the spread of evidence-based and globally harmonized labeling policies, practical implementations have largely focused on FAO and Codex Alimentarius standards. Increasing trade barriers and decreasing cooperation may also influence food labeling policies, focusing on regional food markets. This can stimulate national innovations, with potential (if successful) for regional expansion. A well-documented example of how national policy space can be leveraged to strengthen food labeling in a context of reduced regulatory dependence on global trade norms is provided by South America's adoption of front-of-pack warning labels. Chile was the first country globally to implement a comprehensive mandatory front-of-pack labeling system based on black stop-sign-shaped warning symbols, indicating excessive levels of energy and critical nutrients (47, 48). This policy emerged from domestic public health priorities and was implemented despite notable opposition and concerns regarding trade compatibility. Importantly, the policy was designed as part of a broader regulatory package, including restrictions on marketing to children and limits on school food sales, reinforcing its effectiveness within the food environment. Based on evidence of positive changes even in early phases of the implementation of new rules (i.e, product reformulation by the food industry to avoid warning labels and lower intakes of nutrients of concern) [e.g., (49, 50)], similar warning label systems have been adopted or adapted across the region. This regional diffusion highlights how policy innovation can occur outside traditional global harmonization frameworks, instead spreading through regional learning and public health cooperation. These developments illustrate that de-globalization does not necessarily imply regulatory fragmentation but may also enable policy innovation aligned with national and regional nutrition goals that can then be adopted elsewhere.

Although a substantial body of evidence indicates that interpretive front-of-pack labeling (FOPL) is more effective than traditional back-of-pack nutrition information in influencing consumer behavior and incentivizing product reformulation (51), the adoption of such policies in a de-globalizing context is often constrained by economic and political considerations. However, improvements of food labeling policies are not automatic and depend critically on domestic governance capacity and industry responses. Governments may be reluctant to introduce mandatory nutrition labeling schemes that are perceived to interfere with domestic food industries, affect competitiveness, or disadvantage specific sectors of the local economy. While policy environments oriented toward economic integration may facilitate the adoption of global or regional standards, particularly those aimed at reducing trade barriers, the implementation of health-oriented national policies frequently encounters stronger resistance, especially when they impose regulatory costs or challenge established production and marketing practices. The ongoing debate surrounding the introduction of a harmonized FOPL in the European Union (EU) provides a salient example of such tensions. Despite scientific evidence supporting the effectiveness of interpretive FOPL systems such as Nutri-Score and Food Compass [e.g., (52, 53)], the EU has thus far been unable to reach consensus on a harmonized scheme. Opposition from several EU Member States and industry stakeholders has been grounded in concerns related to national food traditions, potential economic impacts on specific product categories, and perceived trade distortions within the single market (54). This illustrates that even within highly integrated regional blocks, health-driven food policies may be deprioritized when they conflict with economic, cultural, or political interests, underscoring the challenges of advancing nutrition policy in both globalized and de-globalizing governance contexts.

It should be also mentioned that country-of-origin labeling can gain prominence under de-globalization as it can be perceived both as a technical barrier to trade—introducing both additional opportunities and risks. Origin information can shape consumer perceptions of quality, safety, and sustainability and may reinforce trust in domestic food systems, but at same time origin cues may bias consumer perceptions and obscure nutritional quality if not carefully regulated, facilitating misleading “local” or “natural” claims.

## Diversification of diets with plant-based alternatives and indigenous origins

### (Michael Rychlik)

For many reasons, the diversification of diets has been and still is recommended, with plant-based alternatives to animal-derived foods, as well as indigenous foods or ethnofoods becoming increasingly popular. This comes with ethical, nutritional and food safety implications, which will be outlined below.

#### Plant-based alternatives to animal-based foods

Worldwide dietary changes are partly due to consumers questioning the conventional production and consumption of animal-based food for its impact on animal welfare, energy waste, loss of biodiversity, and—last, but not least—for being detrimental to human health. Therefore, plant-based alternatives (PBA) to traditional animal-based foods are increasingly developed, marketed and consumed.

The range of products covers plant-based meat and milk alternatives along with products elaborated from tissues produced by protein-based additive manufacturing.

Apart from their ethical benefits, the nutritional value and safety of PBA have been the topic of recent investigations and disputes.

#### Nutritional value

The sources for PBA are highly variable, and therefore, general conclusions are difficult to draw. However, for their protein components, plant sources show inferior quality compared to animal-based proteins due to their lower content and amino acid composition, except for soy-based alternatives (55). For plant-based milk alternatives, it is also worth noting that real milks are designed to provide all essential nutrients for the growing infant or the respective nursing infant. The detailed differences depend on the plant source in question and for this, soy drinks are generally assumed to be most similar to mammalian milks (55, 56). Other plant-based milk alternatives reveal different and partly severe deficits. The respective PBA made from cereals are generally lower in many vitamins like B12 and B2, minerals such as calcium und other essential elements like iodine (55), unless they are fortified with any micronutrients. Moreover, the drinks made from cereals reveal a much lower ω3 to ω6 ratio (55) in the polyunsaturated fatty acids, which is supposed to be suboptimal.

A significant aspect related to nutrient deficiencies is the prevalence of organically produced and marketed milk alternatives. According to the regulations for organic products in the EU, for these products fortification with nutrients is not permitted (57). Thus, the detrimental lack of nutrients cannot be alleviated, which means that the target of environmentally friendly drinks leads to a lack of nutritional quality.

For plant-based meat alternatives, the plant sources and their processing are even more variable. In addition to the cereals, nuts and legumes used for plant drinks, here oilseeds like sunflower seeds and protein concentrates from soy or wheat (gluten) are applied, even in variable combinations. Moreover, the processing involved is more diverse, including germination or fermentation with bacteria or fungi (Tempeh or Quorn^®^). With these biotechnological processes, the nutritional quality of the plant material is often improved, such as germination or tempeh fermentation, e.g., increasing the content of folates among others (58, 59).

#### Food safety

As microbiological safety is primarily dependent on the hygienic quality of the raw materials and the hygiene of the further processing involved, the following discussion focuses solely on chemical safety.

Among all chemical contaminants, most investigations have focused on heavy metals and mycotoxins. For other contaminants like plant toxins, process contaminants like acrylamide, mineral oil hydrocarbons, or persistent organic pollutants like dioxins or perfluorinated alkyl substances (PFAS), differences may also be assumed, but are less examined and, therefore, will not be outlined here.

For heavy metals and mycotoxins, the relevant compounds can be foreseen from known contaminations in the plant material. Therefore, elevated levels of inorganic arsenic in rice and cadmium and nickel in soy have been confirmed in the respective drinks (60).

For mycotoxins, we developed a new multitoxin stable isotope dilution assay (SIDA) and applied it to a comprehensive market survey of plant-based drinks. In case of oat drinks, we found the estimated daily intake (EDI) of the sum of T2 and HT-2 toxins for toddlers reached 70 % of the tolerable daily intake (TDI) for the mean of all products. For almond drinks, we calculated with the mean aflatoxin B1 content of all products a Margin of Exposure (MoE) for toddlers of below 2,000, which is far below the criterion of alert of 10,000 to initiate risk management actions if undercut. The aflatoxin B1 content was even higher for tiger nut drinks with MoEs being as low as 69 for toddlers. For the emerging mycotoxins produced by *Alternaria* species we found a very common contamination with alternariol (AOH) and its methyl ether (AME). Although there are only monitoring guidelines set by the EU (216), the exceedance of the threshold of toxicological concern (TTC) for the whole set of nut-based drinks would require more toxicological studies and a reduction of the exposure (61). These results are well in line with and extend the recent findings by Torrijos et al. (62) and Gützkow et al. (63), both highlighting the critical occurrence of mycotoxins in plant-based drinks.

The mycotoxin contamination in plant-based meat alternatives was partly similar to that in the milk alternatives. While *Alternaria* toxins were present across all product groups, *Aspergillus* toxins were detected only sporadically, and *Fusarium* toxins were found exclusively in wheat-based alternatives. Seitan products were particularly affected by contamination with both *Fusarium* and *Alternaria* toxins.

The exposure assessment revealed a potential risk, particularly for toddlers, as well as for individuals of all ages, due to *Alternaria* toxins. This risk exists regardless of the product category but is particularly concerning in the case of seitan products.

Therefore, we conclude that seitan products must be systematically monitored for their mycotoxin content in the future, with special attention to *Alternaria* toxins, even though no governmental maximum levels have been established for them yet. In the meantime, these products should not be included in the diet of toddlers.

Although the risk from nutrient imbalance or chemical contaminants in plant-based meat alternatives may not be too severe, milk alternatives are considered more critical. This is particularly relevant when plant-based drinks are used to completely replace breast milk for infants or cow's milk for toddlers. Apart from the lack of important nutrients, which may not even be added to organically produced milk alternatives, oat drinks, almond drinks, tiger nut drinks, and hazelnut drinks may contain mycotoxins that pose unacceptable risks to the latter vulnerable population. This issue must be addressed by business operators through rigorous monitoring of their raw materials, by risk managers through specific maximum levels, and by risk communicators through respective warnings. In general, global trends of transforming diets and food production toward potentially more sustainable alternatives, overlook encountered threats. A proactive approach to foresee possible risks and global monitoring standards for these alternatives need to be established.

#### Indigenous foods

Indigenous Foods or Ethnofoods constitute an important part of the diet of Indigenous populations worldwide and supply them not only with macronutrients, but also with micronutrients (64). Additionally, their content of important dietary phytochemicals offers dietary perspectives also for other regions in the world. With respect to micronutrients, these foods may help in reducing “hidden hunger,” i.e., the lack of micronutrients in an energy-rich diet or “induced hunger,” i.e., by reduced general food intake with glucagon-like receptor 1 (GLP-1) receptor agonist medication (65).

Apart from their nutritive benefits, indigenous foods help sustain nurturing remote communities in current times when global supply chain for foods and thus food security is endangered.

By originating from tropical regions of high biodiversity, many indigenous foods are still analytically under-investigated, economically underexploited or simply unknown to a wider public.

Examples of ethnofood being currently investigated are the Pequi fruit from Brazil (66), or the green plum in Queensland, Australia (67).

Tropical and exotic foods in general hold considerable untapped potential for improving diets, yet the lack of robust analytical data means their nutritional variability remains poorly understood. Local analytical capacity in many producing regions is limited, and conducting analyses in industrialized countries is constrained by international regulations such as the Nagoya Protocol. The intentions of this multilateral agreement are, on the one hand, to prevent the imbalanced exploitation of native resources containing, e.g., pharmaceutically active compounds by multinational companies and, on the other hand, to provide access to these valuable resources by benefit sharing. However, its partially missing implementation hinders obtaining further scientific knowledge on important nutrients.

It requires access and benefit-sharing agreements for nutrient analysis—even when foods are purchased legally in importing countries. In practice, many countries of origin lack clear or timely procedures for granting such permissions, hindering research and sometimes rendering samples unusable before approval is obtained.

Many researchers are unaware of these issues and run the risk of coming into conflict with the laws of their home countries, even though the countries of origin apparently do not always provide the legal authorizations that would be in their own interests. As a result, even one of the authors' publications is currently under threat of being withdrawn (68).

While protecting indigenous communities from exploitation is ethically essential, it can be argued that there is also an ethical responsibility of the countries of origin to avoid obstructing the generation of knowledge that could contribute to solve global nutrient deficiencies.

## Ultra-processed food

### (Mark Lawrence and Barbara Cardoso)

For over 100 years, nutrition science has operated with a predominantly nutrient-centric approach to research and policy activities. The discovery, isolation and elucidation of vitamins and minerals in the first half of the 20th Century led to nutrition policy interventions, successfully tackling many nutrient-deficiency diseases. The emergence of diet-related chronic diseases in the second half of the 20th century was often attributed to excessive consumption of high-fat, salt and/or sugar foods; nutrition policy interventions framed within this context have had mixed success in preventing chronic diseases.

Then, in 2009, an invited commentary challenged nutrition science's nutrient-centric approach by proposing that food processing should be the primary focus of nutrition research and policy activities to prevent diet-related chronic diseases. It introduced the concept of ultra-processed foods (UPFs) (69) as “formulations of ingredients, mostly of exclusive industrial use, that result from a series of industrial processes” (70). They are one of four food groups within the NOVA food processing classification system that categorizes foods according to their extent and purpose of processing: Group 1—Unprocessed or minimally processed foods; Group 2—Culinary ingredients; Group 3—Processed foods; and Group 4—Ultra-processed foods. Examples of UPFs are savory snacks, sugar-sweetened beverages and instant noodles. UPFs contribute over half of total energy intake in the typical diets of both the United States and the United Kingdom (71), and consumption has steadily risen across regions, including Asia, Africa, the Middle East, and Latin America (72).

The UPF concept provided a basis for designing research studies to test the hypothesized relationship between extent and purpose of food processing and diet-related chronic diseases (73). Since the concept's introduction in 2009, scientific interest has surged with almost 20,000 publications referencing UPFs by 2025. Initially dominated by observational studies, this literature consistently links higher UPF consumption to 32 adverse health outcomes spanning obesity, type 2 diabetes, cardiovascular diseases, depression, anxiety, sleep disturbances, and all-cause mortality (74).

While observational data laid the foundation, intervention studies have begun to substantiate causality and elucidate mechanisms. The first *ad libitum* feeding trial, published in 2019, demonstrated that a UPF-rich diet increased energy intake by ~500 kcal/day and led to significant weight gain within 2 weeks (75). Subsequent trials confirmed that even short-term exposure to UPFs can promote weight gain (76–78). Notably, Dicken et al. (79) showed that a UPF-based diet compliant with national dietary guidelines resulted in less weight loss than a minimally processed equivalent, reinforcing the role of processing beyond displacement of whole foods (80).

Emerging trials have begun to unpack behavioral and physiological mechanisms. UPF-rich meals are associated with reduced satiety (81), diminished chewing effort (76), and higher food cravings (79), factors that drive excess energy intake and contribute to weight gain. Further, experimental data also link UPF consumption to dyslipidemia (76, 77) and impaired male fertility (77), suggesting hormonal and metabolic disruption. Parallel animal studies suggest that common UPF additives, such as emulsifiers, preservatives, and colorants, as well as processing by-products like acrylamide and advanced glycation/lipoxidation end-products, contribute to gut microbiota dysbiosis, reduced short-chain fatty acid production, increased intestinal permeability, and chronic inflammation (82–84).

The consumption of UPFs also presents a critical barrier to protecting planetary health, although their environmental impacts remain poorly explored. The 2025 EAT–Lancet Commission on healthy, sustainable, and just food systems reported that current dietary patterns are major drivers of planetary boundary transgressions, contributing to climate change, biodiversity loss, freshwater depletion, and nutrient pollution across global food systems (85). UPFs contribute to the negative global environmental change, since they contribute disproportionately to land degradation, biodiversity loss, and greenhouse gas emissions, particularly through the production of processed meats and high-demand plant-based ingredients like palm oil and soy (86). Their manufacture relies heavily on industrial agriculture, which intensifies fertilizer use and water consumption, also driving aquatic ecosystem disruption (86). The widespread use of novel entities in packaging and processing, such as plastics and synthetic additives, adds further complexity to sustainability challenges, yet remains critically understudied. Beyond ecological concerns, UPFs are embedded in highly concentrated food systems dominated by a few transnational corporations, whose control over agricultural inputs, commodity trade, and retail pricing shapes food access and equity (87).

These multiple evidence streams have further strengthened the validity of the hypothesized relationship between the extent and purpose of food processing and diet-related chronic diseases. Consequently, there are increasing calls to extend the scope of UPF research (88) and to translate the rapidly growing body of evidence into UPF policy (89). The quality of UPF-related research and policy will be strengthened by improving the design of food surveillance tools, such as food availability, purchasing and consumption surveys, to enable the collection of comprehensive and accurate UPF data. Similarly, food composition databases will need to provide more information on *chemical* markers of UPFs such as cosmetic additives to assist with UPF identification. The use of large language models in sourcing and categorizing these data will likely become more common. Accompanying the development of these novel UPF-related food composition databases could be an exploration of the development of innovative databases on *physical* markers of UPFs. Such databases would be designed to provide information on the use of processing aids and processing technologies such as extrusion, high pressure and high temperature, that affect the physical structure of a food matrix during the manufacture of food products. Meanwhile, integration of poly-metabolite scores predictive of UPF intake with self-reported dietary data offers promise for refining exposure assessment and strengthening causal inference (90).

To date, most UPF trials have focused narrowly on weight-related outcomes. Future intervention studies need to broaden their scope to investigate the full spectrum of diseases identified in observational research, including mental health, sleep, respiratory, and cardiometabolic outcomes. Drawing on evidence from these observational and experimental studies to quantify the proportion of the global and national burdens of disease attributable to UPFs will be important for raising the UPF concept as a priority for food policy and regulatory actions. The next phase of UPF research needs to prioritize mechanistic clarity, translational relevance, and policy impact, ensuring that nutrition science continues to evolve from association to actionable insight.

Into the future a priority consideration for food and nutrition policymakers will be to reform how they inform nutrition policy reference standards, such as national dietary guidelines, to incorporate recommendations to reduce UPF consumption (91). The translation of these dietary guideline recommendations into practical advice represented in national food selection guides will require replacing the current vaguely termed “discretionary” food group with the evidence-informed term “ultra-processed food” group (92). These dietary guideline and food selection guide recommendations could be readily applied to the designs of food policy actions intended to promote the substitution of minimally processed foods for UPFs. For example, UPFs might be required to display a front-of-pack warning label. Also, policy decisions on allocating fiscal measures such as food taxation and subsidies, marketing and/or food procurement restrictions might be based on whether a food is categorized as a UPF (Nova group 4 food) or a minimally processed, culinary ingredient or processed food (Nova group 1–3 foods).

The UPF concept represents a major nutrition science innovation. It helps operationalize the reorientation of nutrition science from its conventional nutrient-centric approach to a primarily food processing approach. This reoriented nutrition science is inclusive of the biological, social and environmental dimensions of food and health associations, and so it is more relevant to contemporary nutrition problems. Professional nutrition associations are also increasingly engaging with the UPF concept, notably in 2025 the International Union of Nutritional Sciences established a UPF Task force for the period 2025–29. The next 5 years will be an exciting period for UPF-related research and policy to support the substitution of minimally processed foods for UPFs to promote healthy, equitable and sustainable diets.

## A framework for plastics in food

### (Johannes le Coutre)

#### Micro-and nanoplastics in food systems

Plastics are among the most transformative inventions of the twentieth century, offering durability, versatility, and low production costs. Yet these same properties have also produced a legacy of persistent plastic waste, which fragments into microplastics (MPs, 1μm-5 mm) and nanoplastics (NPs, < 1 μm). Collectively, these micro- and nanoplastics (MNPs) are now recognized as ubiquitous pollutants that contaminate terrestrial, freshwater, and marine ecosystems.

Food systems are at the center of this problem. Agriculture uses plastics extensively in mulching, irrigation, packaging, and greenhouses. Aquatic systems are sinks for plastic waste, with seafood serving as a dietary vector. Food processing and packaging contribute further exposure, and airborne microfibers can contaminate prepared foods. As a result, humans are exposed to MPs on a daily basis, with recent studies confirming their presence in blood, lungs, placenta, stool (93), and even brain (94) and bone tissue (95).

Despite this growing evidence, major uncertainties remain about the scale of risk. While *in vitro* and animal studies demonstrate oxidative stress, inflammation, and genotoxicity, direct epidemiological data linking MNP exposure to human disease remain sparse. At the same time, food process engineering presents overlooked opportunities: unit operations such as filtration, centrifugation, spray drying, and enzymatic treatment may either contribute to MNP enrichment or provide avenues for mitigation. This section synthesizes the current state of knowledge, focusing on sources, composition, health effects, and the underexplored engineering interventions that could help safeguard future food systems.

#### Sources and pathways of contamination

The entry of MPs into food systems is both diffuse and systemic, encompassing agriculture, aquaculture, packaging, and domestic practices.

*Agricultural Inputs:* The Food and Agriculture Organization (FAO) has identified agriculture as a major vector of plastic contamination. Plasticulture—plastic-based agricultural practices—has expanded globally since the 1950s, especially in intensive systems such as those in China, Spain, and the Middle East (96). Plastic mulch films, widely used to conserve water and suppress weeds, fragment under ultraviolet light and mechanical abrasion, generating MPs that accumulate in soils. In some Chinese croplands, mulch residues exceed 500 kg per hectare, where they interfere with soil structure and microbial activity.

Sewage sludge and compost, both used as fertilizers, introduce further plastic loads. Wastewater treatment plants remove most visible plastics but release high volumes of small MPs and NPs that are retained in biosolids. When these are applied to fields, they become long-term reservoirs of contamination. Irrigation with wastewater and atmospheric deposition of airborne fibers from urban and rural environments compound the problem.

Crops themselves can internalize MPs. Studies have shown polystyrene and polyethylene nanoplastics entering root systems and translocating to aerial tissues in lettuce, wheat, and rice (97). Foliar uptake through stomata has also been observed, raising the likelihood that edible plant tissues harbor internalized plastic particles. This represents not only a contamination route but also a disruption of plant physiology, a s discussed later.

*Aquatic Systems:* Aquatic environments act as both transport routes and sinks for plastics. Rivers carry urban and agricultural plastics into oceans, where fragmentation creates abundant MPs. Marine organisms ingest these particles, which bioaccumulate in tissues consumed by humans. Bivalves such as mussels and oysters are particularly susceptible because of their filter-feeding habits. Fish and crustaceans also ingest MPs, and microplastics have been consistently detected in seafood products (98). Drinking water represents another direct dietary source. Tap water samples in multiple countries contain plastic fragments, and bottled water is often more contaminated. One study reported that a liter of bottled water can contain up to 240,000 particles, primarily PET and polyamide (99). These findings underscore that MNP contamination is not limited to polluted environments but also occurs in consumer products widely considered safe.

*Food Processing and Packaging:* Processing and packaging are additional routes of exposure. Plastics are widely used in food contact materials, and degradation during heating, abrasion, or storage releases particles. Polypropylene containers shed thousands of particles when microwaved. PET bottles release MPs during reuse and prolonged storage. A dietary intervention study showed that individuals consuming highly packaged foods had higher stool microplastic concentrations (93).

Cooking practices also contribute. brewing tea in plastic-sealed tea bags can release tens of thousands of particles per cup, while using plastic kettles or microwave containers exacerbates leaching. The domestic kitchen thus represents another point of exposure.

*Atmospheric Fallout:* Airborne fibers and dust settle on exposed foods. Indoor air has high concentrations of synthetic fibers from textiles and furnishings, which contaminate meals during preparation and serving. Inhaled fibers can also be transported via mucociliary clearance to the gastrointestinal tract, indirectly adding to ingestion. This atmospheric pathway means that even unprocessed and unpackaged foods are not exempt from MNP contamination.

*Composition and Properties of MNPs:* MPs are highly heterogeneous. Common polymers identified in food systems include polyethylene (PE), polypropylene (PP), polystyrene (PS), polyvinyl chloride (PVC), polyethylene terephthalate (PET), and polyester fibers (93, 97). These appear in various shapes, including fragments, films, foams, beads, and fibers. Their size distribution ranges from visible MPs down to nanoscale particles capable of crossing biological membranes.

Surface properties are central to their environmental behavior and toxicity. Weathering through UV exposure, oxidation, and mechanical abrasion increases surface roughness and hydrophilicity, enhancing sorption of heavy metals, pesticides, and organic pollutants. Many plastics contain chemical additives such as bisphenol A, phthalates, and flame retardants, which leach during degradation and act as endocrine disruptors.

Nanoplastics deserve particular attention. Their high surface-to-volume ratio increases reactivity and facilitates cellular uptake. They can penetrate cell membranes, accumulate within organelles, and catalyze the formation of reactive oxygen species. These properties make them more biologically active and potentially more harmful than larger MPs (98).

#### Environmental contamination, photosynthesis, and biosequestration

The impacts of MNPs extend beyond food safety to fundamental ecosystem processes, especially photosynthesis. In plants, nanoplastics can penetrate root tissues and translocate to leaves, where they interfere with chloroplast function. Studies have shown reduced chlorophyll content, inhibited photosystem Il efficiency, and lower biomass in crops such as lettuce, rice, and wheat exposed to NPs (97). These disruptions impair photosynthetic capacity and crop yields.

Aquatic photosynthetic organisms are also affected. Microalgae and cyanobacteria exposed to microplastics exhibit reduced photosynthetic efficiency, lower chlorophyll content, and altered carbon fixation (98). In some cases, shading effects from plastic particles in the water column reduce light availability, further inhibiting productivity. Because algae form the base of aquatic food webs, these effects can cascade through ecosystems, affecting higher trophic levels and fishery yields.

Soils and plants also act as reservoirs of plastics through biosequestration. Soil aggregates can trap MPs, reducing their mobility but embedding them in long-lived structures. Plant roots can accumulate particles in the rhizosphere, and biofilms readily colonize plastic surfaces. While biofilms may promote microbial degradation of plastics, they also risk serving as vectors for pathogens and antimicrobial resistance genes (100). Thus, biosequestration stabilizes plastics in ecosystems but simultaneously prolongs contamination.

Agricultural contamination is now widespread. Plastics have been documented in farmlands across Asia, Europe, and the Middle East, with growing concerns in Africa and Latin America as plasticulture expands (96). Given the global reliance on plastic-based agricultural inputs, future food systems are likely to remain exposed unless alternative materials and collection systems are developed.

#### Human health risks

Evidence of human exposure is clear. Microplastics have been detected in stool, blood, lungs, placenta, breast milk, and even in the brain and bone tissue (95). Humans may ingest tens of thousands of particles annually through food, beverages, and air.

Health concerns are increasingly supported by clinical findings. Italian researchers identified microplastics in carotid artery plaques, correlating their presence with a 4.5-fold higher risk of major cardiovascular events (myocardial infarction, stroke, or death) over approximately three years. Some *post-mortem* studies report higher brain micro-/nanoplastic burdens in neurodegenerative disease cohorts, but causality is unproven and replication is needed. Chinese researchers reported microplastics in bone and skeletal muscle, raising questions about musculoskeletal health and exercise capacity (101).

Mechanistically, plastics are linked to oxidative stress, lipid peroxidation, DNA damage, and chronic inflammation. Nanoplastics cross membranes and accumulate in organelles, disrupting cellular metabolism. Some evidence suggests plastics accelerate aging by impairing vascular function and maintaining low-grade inflammation. Others speculate about links to cancer, particularly colorectal cancer, given high stool microplastic loads (97).

Despite these findings, researchers stress that MPs are unlikely to act as sole causes of disease. Rather, they may act synergistically with other environmental and lifestyle factors, amplifying chronic disease risks. Unlike asbestos, which directly causes mesothelioma, MPs contribute indirectly by increasing cellular stress and weakening resilience. Epidemiological data remain sparse, underscoring the need for more longitudinal human studies.

#### Process engineering opportunities for mitigation

While upstream reforms in agriculture and packaging are essential, food process engineering could provide immediate, practical interventions.

Filtration and membrane technologies are among the most effective. Microfiltration, ultrafiltration, and reverse osmosis, already used in beverage industries, can retain MPs and are effective in water treatment. Applying them more broadly in dairy, juice, and brewing could substantially reduce consumer exposure.

Centrifugation and sedimentation exploit density differences between plastics and water. Since PE and PP are less dense than water, separation systems could be designed to float and remove them. Conversely, denser polymers such as PET may be concentrated in sediment fractions for removal.

Adsorption and clarification, already employed in winemaking and brewing, could also be extended. Activated carbon, bentonite, and biochar have strong affinities for hydrophobic surfaces and could immobilize plastic particles. Research is needed to optimize dosages and operational conditions for microplastic removal.

Spray drying may also provide a novel route. Because it involves atomization, rapid dehydration, and airflow control, spray drying may influence particle separation or concentration in powdered foods (102). Preliminary evidence suggests it could alter the distribution of contaminants, potentially enabling MNP reduction in milk powders, infant formula, or protein powders. This remains speculative but merits systematic exploration.

Biological and enzymatic treatments are emerging. Enzymes such a s PETase and engineered cutinases can degrade PET and other polymers under mild conditions. Their integration into wastewater treatment in food plants could reduce MNP recirculation (103, 104).

Material substitution and process redesign are critical. Replacing plastics with stainless steel, glass, or biodegradable polymers in processing lines minimizes particle shedding. Avoiding hot-filling or microwaving in plastic reduces fragmentation. Hygienic closed systems can reduce airborne deposition.

Finally, monitoring and governance must evolve. Spectroscopic techniques such as FTIR and Raman spectroscopy, together with pyrolysis-GC/MS, are available to identify and quantify MNPs (105). Embedding these methods into Hazard Analysis and Critical Control Point (HACCP) frameworks would formalize plastics as recognized food safety hazards, allowing proactive control alongside microbial and chemical hazards.

Micro-and nanoplastics are deeply embedded in food systems, from soils to seafood and from packaging to the kitchen table. Their chemical diversity and persistence ensure long-term contamination, while their capacity to disrupt photosynthesis and their embedding in soils highlights risks for agricultural productivity and ecosystem function. Agricultural contamination is now widespread and systemic, underscoring the urgency for action.

Although uncertainties remain regarding direct human health effects, evidence increasingly suggests that MNPs act as chronic stressors, exacerbating cardiovascular, neurological, and oncological risks. The food industry occupies a pivotal role: while contributing to exposure through packaging and processing, it also holds powerful tools for mitigation.

Process engineering interventions—including filtration, centrifugation, adsorption, spray drying, enzymatic treatments, material substitution, and monitoring—offer immediate pathways to reduce exposure. Embedding plastics into HACCP frameworks and aligning downstream interventions with upstream reforms are essential for long-term resilience.

Addressing MNPs requires a holistic approach spanning environmental management, agricultural reform, food engineering, and public health. By leveraging engineering innovation and systemic reform, food systems can transition from passive vectors of contamination to active participants in mitigation, thereby protecting both human health and ecological stability in the plastic age.

## BIG data and the arrival of comprehensive food composition

### (J. Bruce German)

#### Big data

The term is revolutionizing fields of science from genomics to astronomy. Nutrition has been behind but that is all about to change. This new era of scientific inquiry and translation didn't just arrive overnight. It is ostensibly the combination of several enabling breakthroughs. First was conceptual, the vision that computational machines could learn and build upon prior calculations (106). The second was technological as the world first took note of the high-capacity computer server networks capable of storing and accessing truly large datasets (107). This capacity to store large datasets propelled fields to very large quantities of digital data for mathematical interrogation was emerging. Various fields began to rethink experimentation and data collecting with the promise that computational storage capacity was no longer limiting. The rapid development of massive (super) computing power made it possible to take the final step, algorithms that bring together large datasets with machine learning (108). The public were convinced of the power of AI by large language models and face recognition, but scientists were astonished by the breathtaking revelations in biology. When AlphaFold made it possible to take the sequence of a protein in one dimension and solve for its solution structure at angstrom resolution in three dimensions, there could be no denying, AI is among the most consequential recent advances in computational capability, with clear implications for nutrition research and practice (109). But nutrition was being left behind, no big data.

Nutrition was being left behind by the explosion of AI for the most basic of reasons, it lacked its most important big data: the precise chemical composition of food (110, 111).

If scientists wished to solve for what diet does to human health, it is necessary to know what food is. Of the thousands of chemicals in the thousands of foods around the world, databases contain a tiny fraction of that critical data. Yet the tools to acquire such information are now emerging and led by several key initiatives, notably the Periodic Table of Foods Initiative (https://foodperiodictable.org/), also the USDA database (110) and Metrofood-RI (111), the accurate, quantitative databasing of the world's food is arriving (112). What will this mean to the field of nutrition?

#### Pattern

The heroic achievement of nutrition was the discovery of the essential nutrients. Of the hundreds of thousands of biological chemicals in the environment, scientists in the 20th century identified all of the relatively few vitamins, minerals, fatty acids and amino acids that are essential. This is a magnificent achievement of science by any criteria. It is also important to recognize how those discoveries were made. The discovery of the essential nutrients is a triumph of reductionist science. The strategy of purifying chemicals in foods and identifying those individual molecules whose explicit removal from the diet resulted in frank deficiencies was critical. However, the success of reductionist strategies for the essential nutrients has left a legacy of expectation that all chemicals act individually within the diet. The emergence of non-communicable chronic diseases linked to dietary patterns has highlighted the complexity of diet and health. That apparent complexity has not discouraged scientists from continuing to pursue reductionist experimentation. What is the one macromolecule causing or preventing heart disease, what is the one dietary ingredient that alters inflammation? Those studies have been at best discouraging. Observational studies have stated for decades that dietary patterns are associated with the diversity of health outcomes on different diets in different environments for different subjects (113). Now, those patterns can be resolved. The first benefit of food compositional databases and the computational AI tools to interpret them is conceptual. Nutrition will become an integrative, multi-factorial, pattern recognition science. Just as the 20th century reductionist science guided the discovery of each of the chemicals associated with scurvy, anemia, goiter, etc., the integrative tools of the 21st century will see the patterns of diet responsible for insulin resistance, hypertension, inflammation, etc.

The detailed, annotated knowledge of diet through accurate compositional mapping at its core will have far reaching benefits to society, just as its lack has had far reaching costs. The entire agricultural enterprise will be linked through its chemical and biological outputs. We will know what diets are most appropriate for early childhood, pregnancy and lactation, aging, for protection from disease and recovery from injury, for performance and delight, locally, safely and affordably. It will be possible to manage agriculture as foods' chemical inventory matching supply and demand in real time. Efficiency of agriculture will increase dramatically freeing land and water for rewilding. The chemical and biological knowledge of agricultural production, food processing and dietary management can infuse greater value into each of these massive global activities and assist with income inequality across the food system. The field of nutrition will be transformed into a big data, integrative science delivering precision solution across the entire agriculture and food enterprise. However, to reach these opportunities, this generation has important challenges.

#### The data

Transformative foodomics analytical tools, the computing power to accumulate massive datasets and the computational tools to interpret them are arriving. Complete food composition data will need equally inspiring experimental and clinical designs. Now the challenge is to initiate studies that can use these tools to maximum effect. Outcomes from Framingham was a bold and inspiring example that informed science for a half century (114).

What subsets of the population should be nutrition's priority? What outcomes will lead to mechanistic insights for health more generally? Serum markers of cholesterol and glucose have informed public health for a century; is it time to broaden outcomes to include all of metabolism, immunity and physiology as well as essential nutrient status? Context. What metadata will provide the context for diet and health? Modern agriculture is built on the goal of quantitative abundance: just grow more. As agriculture shifts to a quality-based value proposition, what is quality for health? Food processing has focused on safety and organoleptic preference. What do the various unit operations in food processing do to the quality of foods as the health of their consumers? Why do individuals choose particular foods to make up their daily diets? Instinct apparently doesn't work for humans as throughout history we have eaten diets that while delicious and satisfying were deficient and chronically toxic. Why don't we all prefer healthy diets? What data must we collect to inform the answers to those questions?

#### Education

A transformation of the entire agriculture and food sector catalyzed by precise compositional databases of food and computational tools of AI will require a new approach to educating the next generation of scientists. The reductionist model of investigation, so successful for the essential nutrients, is giving way to a complex multifactorial approach, that addresses major scientific challenges from increasing the efficiency of agriculture to delivering precision nutrition. Educational models must include the tools of data management from knowledge maps to ontologies and computational pattern recognition using examples from nutrition, from the complexity of food to the diversity of humans. As just one example, the challenges to understand and improve the functions of the human intestinal microbiome highlight this complexity. The microbiome consists of trillions of bacteria in communities of hundreds of species, actively competing for hundreds of accessible substrates and in turn releasing hundreds of metabolites to the host. No single bacterium, carbohydrate or metabolite is responsible for controlling the complex functions of this community to the overall nourishment of the host. This ecosystem must be understood as an ensemble of interactive variables.

The challenge to educating the next generation of nutrition students as a key to transforming the entire agriculture and food system is even more urgent because of scale. Many modern innovations can be scaled rapidly because the key innovation is identical everywhere. The same mobile phone that works in San Francisco, works in Johannesburg. That simplifying uniformity cannot work with food. The key to making agriculture stable and efficient as a food supply is recruiting biological diversity around the world. Attempting to grow identical crops everywhere has proven unsustainable. Different ecosystems in different environments must be addressed locally. The nutrition community will need to be educated globally to examine the diversity of potential crops to identify those that succeed by multiple criteria, locally. The Periodic Table of Foods Initiative has begun such an ambitious educational vision with their FoodEDU program (115).

With the arrival of such enabling tools to propel how we do scientific discovery, it is a time to assemble the consensus wisdom on why we do scientific discovery. In the beginning of this new era is it time for nutrition to re-prioritize the targets of knowledge discovery? As we educate the next generation of young scientists, can we inspire them with a new breadth and depth of targets that can improve the human condition. Is it, for example, time to prioritize the health, development and performance of mothers and infants, is it time to understand the metabolic, physiologic and neurologic determinants of spontaneous physical activity, is it time to prioritize the mechanisms underlying food preference and the pathologies of eating disorders?

## *One Health* impact on food science and nutrition

### (Elena Ibáñez and Alejandro Cifuentes)

*One Health* is an integrated, unifying approach that aims to sustainably balance and optimize the health of people, animals and ecosystems. It recognizes the health of humans, domestic and wild animals, plants, and the wider environment (including ecosystems) are closely linked and inter-dependent. Below, we discuss how the implementation of *One Health* will impact the Food Science & Nutrition fields. Basically, generating a shift from narrow, human-centric and biomedical focus to a wider definition focusing on whole society and how the anthropogenic changes will impact ecosystem and planetary health.

The United Nations SDGs (37) are strongly linked to the concept of One-Health as they include targets for health and wellbeing, clean water and sanitation, climate action, as well as sustainability in marine and terrestrial ecosystems. The One-Health concept transcends anthropocentrism, attempting to simultaneously provide optimal health for humans, animals, and the environment, following a sustainable development (116).

The impact of *One Health* on Food Science needs to be explored since it is expected that *One Health* originates a profound change in many of its basic concepts and approaches. In this regard, the One-Health High-Level Expert Panel (OHHLEP) (117) [i.e., the scientific and strategic advisory group to the Quadripartite organizations—the Food and Agriculture Organization of the United Nations (FAO), the United Nations Environment Programme (UNEP), the World Health Organization (WHO) and the World Organization for Animal Health (WOAH)] has defined a group of underlying principles that may impact on our current concept of Food Science; among them, the responsibility of humans to change behavior and adopt sustainable solutions that recognize the importance of animal welfare and the integrity of the whole ecosystem, thus securing the wellbeing of current and future generations.

Food production is the primary driver for breaching five of the planetary boundaries and accounts for around 30 percent of global greenhouse gas emissions, but still billions of people lack access to healthy diets. The last EAT-Lancet Commission report of 2025 shows that shifting to the *Planetary Health Diet* could prevent up to 15 million premature deaths per year and transforming food systems could cut these emissions by more than half, being a crucial point the reduction of food loss and waste. The EAT Lancet Commission stresses that transformation requires bundled policy measures—such as subsidies for fruits and vegetables combined with taxes on unhealthy foods—alongside stronger social protections to ensure a just transition (85). Other important findings of this report are that fewer than 1% of the world's population is currently in the “safe and just space,” where people's rights and food needs are met within planetary boundaries and that the wealthiest 30% of people drive more than 70% of food-related environmental impacts. Across all regions, the report reveals a common shortfall: diets consistently lack sufficient fruits, vegetables, nuts, legumes, and whole grains. In many places, the analysis also finds that diets contain excess meat, dairy, animal fats, sugar, and excessively processed foods. Building on existing data, the 2025 Commission has strengthened evidence of the benefits of the Planetary Health Diet, which sets out recommendations for healthy diets that ensure nutritional adequacy, support optimal health outcomes, and can be adapted to different contexts and cultures. It emphasizes a plant-rich diet, with optional, moderate amounts of animal-source foods and limited added sugars, saturated fats, and salt. There is also good evidence that adoption of diets in line with the Planetary Health Diet would lower the environmental impacts of most current diets. These targets are directly related to the ones highlighted by the last report from The Lancet *One Health* Commission.

Namely, The Lancet *One Health* Commission published its report on this matter in August 2025 (118). Interestingly, *One Health* proposes to carry out all these changes considering the symbiotic relationship between humans, animals, and the environment that we share, since an expanding array of interlinked threats to humans, animals, plants, and a myriad of other biotic and abiotic elements in our shared ecosystems has been generated. Health challenges such as climate change, antimicrobial resistance, non-communicable diseases, and food insecurity highlight the inextricable interconnections between human, animal, and ecosystem health and reveal the urgent need for a *One Health* approach to informing and implementing solutions. Of utmost importance from a *One Health* perspective is the question of how to sustainably and equitably meet the food and nutrition needs of a growing, more affluent, and socially dynamic human population, while promoting the health and wellbeing of humans, animals, plants, and the environment at large. Interestingly, in the mentioned report there is not discussion about how these changes will affect the Food Science & Nutrition field. Of course, comments can be found related to the expected effects in food systems, food security and food safety, with special emphasis on targeting equitable, sustainable, and healthy food systems via *One Health*. In this regard, it is interesting to mention that food systems encompass all actors and activities involved in all stages of food production, processing, distribution, consumption, and disposal. Food systems span agriculture, fishing, and forestry and operate at local, national, regional, and global levels. Also, the report provided by The Lancet *One Health* Commission mentions that Food systems are largely extractive and destructive, reinforcing inequities and generating and perpetuating an unsustainable, unsafe, and unhealthy range of environmental, health, and socioeconomic costs linked to food. These costs (nearly US$12 trillion annually) exceed the value of the global food systems output. Achieving equitable, sustainable, and healthy food systems entails addressing several crucial challenges: foodborne diseases, undernutrition and overnutrition, and unsustainable trends in agri-food systems. Moreover, the challenge of sustaining the fundamental functions of global food systems, including food security and safety, nutrition, livelihoods, social security, and economic development, while minimizing the negative impacts on global public goods, such as climate stability, natural resources, ecosystem services, biodiversity, and public health, remains a *One Health* priority.

The links among agriculture, food and feed production and biodiversity loss are strong and show a 69% biodiversity decline since 1970, primarily driven by land-use change for agricultural production. Besides, nitrogen-rich nutrient run-off from aquaculture and terrestrial agriculture cause eutrophication and lethal algal blooms contributing to biodiversity loss. Altogether cause an increased likelihood of communicable disease at the wildlife-livestock-human interface, with abundance of reservoirs host species in human-dominated landscapes relative to biodiverse ecosystems. The impact on Food Science is seen via an increased likelihood of non-communicable diseases induced by homogenization of diets, reduced crop yields, increased reliance on fewer crop and animal species for food provisioning.

**Figure 2:**
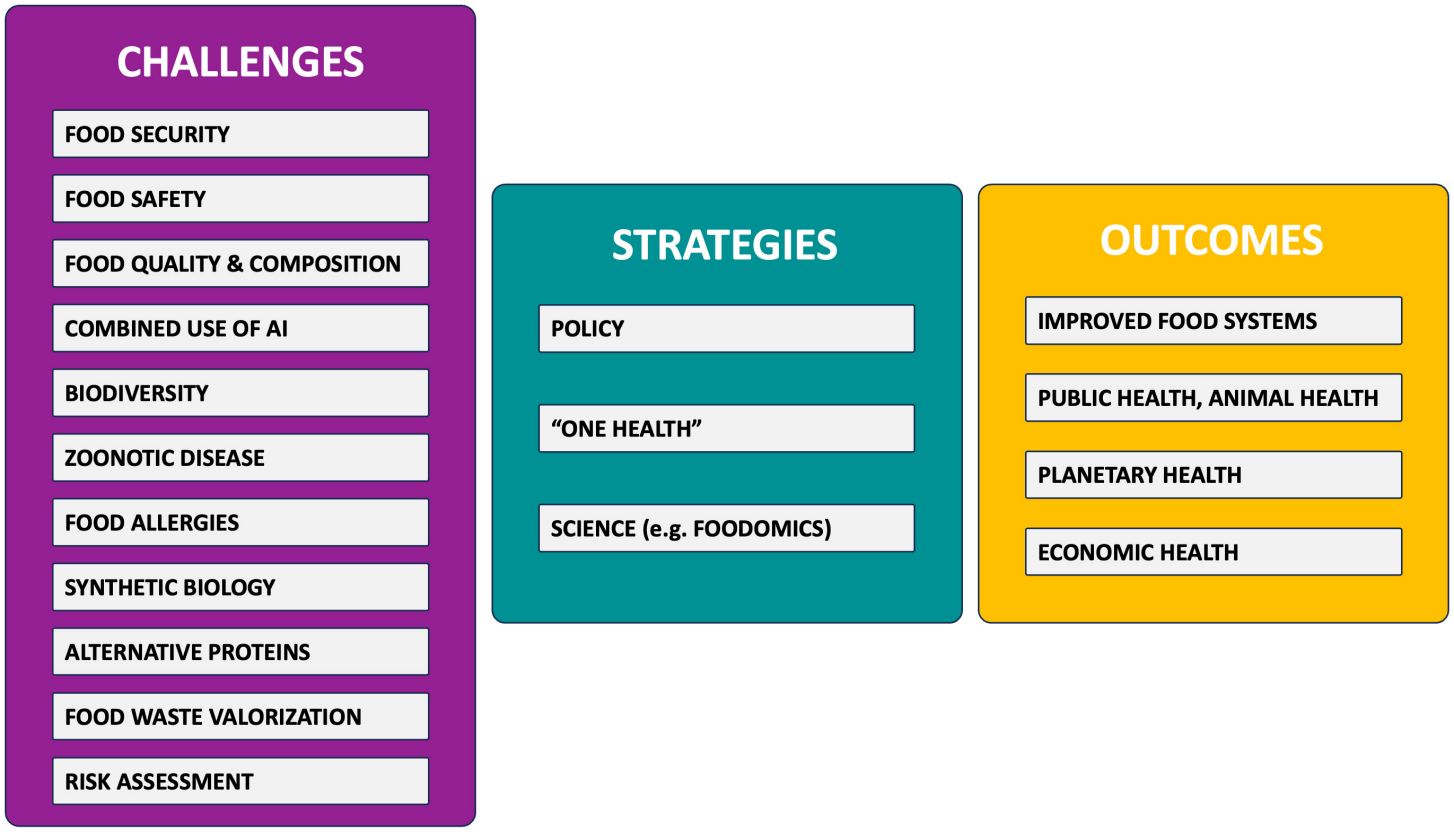
The “*One Health*” approach with core elements and priorities. Economic Health has been included as an additional dimension.

In summary, *One Health*, although formulated two decades ago, remains challenging to implement, even when it may help to solve many of the planetary challenges that we have still to face (see Figure 2), including an important improvement of our knowledge of food systems, planet sustainability and combined health of humans, animals and environment. As an example, the manuscript by Comi et al., shows a clear link between the SDGs and One-Health through the selection of the most interesting agro-food wastes as supply chains for producing nutraceuticals (119). Important aspects to be considered here are: the supply chains should be committed to sustainable practices, with an appropriate bioactive content and potential activity against the target disease, making use of possible innovative and sustainable technologies to obtain the active ingredients, the fulfillment of the Agenda 2030 and One-Health principles, the geographical proximity and certificates of commitment to environmental stewardship, quality control and social responsibility.

Through the implementation of *One Health*, important impacts in Food Science & Nutrition will be observed, e.g., the inappropriate use of certain antimicrobials, pesticides and insecticides; the understanding of the role of the microbiome on different diseases; the discovery of complex foods/products composition; the improvement in genetic diversity and biosecurity; the investigation of zoonotic infections (including, e.g., pathogenic pathways, transmission dynamics, diagnostic biomarkers and novel vaccines in prion, viral, bacterial, protozoan and metazoan zoonotic infections); the investigation of antimicrobial resistance (AMR) (including the mechanisms involved and the discovery of novel treatments); the detection of allergens, exposure of adulteration, identification of pathogens and toxins; the finding of new alternative protein sources; the improvement of food quality, safety and security at global scale; the revalorization of food wastes through new and sustainable processes, and the transformative potential of AI combined with *One-Health* for disease surveillance and health-care delivery.

In the following, we describe some important challenges identified and shared between One *Health* and Food Science & Nutrition that are related to:

1. *Food access and diets:* Paradoxically, even as more than 2 billion people face food insecurity, the world is also grappling with a growing burden of excess weight: in 2022, an estimated 2.5 billion adults were overweight, including 890 million living with obesity, and prevalence continues to rise. We will need to close a significant food-gap by 2050 due to continued population growth and changing diets. In this regard, a recent editorial published in Nature highlights the problem of malnutrition of children. Namely, the aim of eliminating malnutrition among children under the age of five might not have been reached by the SDGs' 2030 deadline, although it seemed achievable. That is no longer the case. An authoritative report called the Joint Child Malnutrition Estimates, compiled by the UN children's organization UNICEF, the World Health Organization and the World Bank, confirmed what many healthcare professionals around the world already suspected (120): since 2020, there has been an increase in the proportion of children under five affected by stunting (121).
2. *Food loss and waste:* more than one third of all food produced globally is wasted (217), along with all the resources needed to produce it, including labor, land, water, and energy.
3. *Food safety:* Despite continuous investment, the WHO estimates 600 million (almost 1 of 10 people in the world) cases of foodborne illness and 420,000 deaths every year (5,000 in Europe).
4. *Agri-food production*: including manufacture, food preparation and cooking, accounts for approximately 30% of all greenhouse-gas emissions and livestock production accounts for approximately 50% of this.
5. *Food composition:* only 150 nutritional components are tracked in food composition tables by the United States Department of Agriculture and other national databases—about 0.5 percent of the 26,625 chemical compounds documented in food; this provides a very poor representation of this complex issue.
6. *Sustainable food systems:* There is a need to optimize the affordable production of safe and nutritious food, while simultaneously supporting livelihoods and maintaining public goods, such as ecosystem services, reduced greenhouse gas emissions, and healthy human and animal populations.
7. *Democratic, inclusive and healthy food systems*: Maldistribution of power within food systems (reducing resilience against shocks and producing food shortages employed as a war tool), globalization (reducing biodiversity, and making more difficult access to local foods and nutritional habits), trade liberalization, increased foreign direct investment in and financialization of the food sector, and pervasive and aggressive promotion of ultra-processed foods and unhealthy eating habits all make highly processed and unhealthy foods widely available and affordable on domestic markets, which undermines public health.

As the EAT–Lancet Commission suggests (118), a joint action plan for equitable, sustainable, and healthy food systems should be established and implemented at the international level and translated at the national level. Implementation of this plan should take place through a process of equitable collaboration and policy integration across all relevant sectors and at global, regional, national, and subnational levels. The joint action plan should address key food systems challenges, including the triple burden of undernutrition, overnutrition, and micronutrient deficiency; zoonotic and foodborne diseases; antimicrobial resistance; greenhouse gas emissions and climate change; and environmental degradation and destruction driven by agricultural expansion. The *One Health* approach to food systems should:

1. Challenge the prevailing corporate systems that drive inequitable, unsustainable, and unhealthy production and consumption trends
2. Ensure that the right to adequate food and food security are achieved in tandem with food sovereignty (i.e., sustainable and equitable food production and consumption systems, in which power lies with local producers and consumers)
3. Recognize the crucial role that the informal food sector plays in food security and livelihoods, as well as the need to support safer food production and exchange among informal producers and market stakeholders
4. Critically examine the financialization of the food sector and trade liberalization, and resist the pervasive and aggressive promotion of inexpensive, highly processed, and unhealthy foods
5. Hold industry and the private sector accountable for the health and sustainability risks that they bring to the food system, including by requiring the disclosure of environmental, social, and governance risks, to enable assessment and informed decisions among investors
6. Advance in the production of healthy, high-quality food systems that, among other things, reduce food waste by half and promote a dietary transition in service to sustainability and health

In conclusion, as global environmental pollution increases, climate change worsens, and population growth continues, the challenges of securing a safe, nutritious, and sustainable food supply have become enormous, as well as the expected impact on Food Science & Nutrition. This has led to new requirements and trends for future food supply methods and functions. Clearly, we need climate-smart and environmentally sustainable and safe food systems linking biorefinery and bioeconomy. Global application of the *One Health* model can ensure future food supply by reducing the effects of human activity on environment and planetary boundaries, enhancing health and wellbeing of humans, animals and the ecosystems we cohabit. Still, we have a long way to go till we are able to understand all the impacts in Food Science that *One Health* will bring about when routinely applied but this impact will foreseeably include a strong interconnectedness humans-animals-ecosystems for a more equitable, sustainable, and healthy planet.

## Precision nutrition: current landscape and future directions

### (Annalisa Terranegra and Angela Zivkovic)

Precision nutrition aims to develop interventions that prevent or treat chronic diseases based on individual traits including genetics, race, gender, health history and lifestyle habits. It recognizes that people may respond differently to specific foods and nutrients, as emphasized by major institutions such as the Harvard T.H. Chan School of Public Health, the National Institutes of Health (NIH), and the Academy of Nutrition and Dietetics (AND) (122–132). When developing an individualized diet, multiple factors must be considered, including demographic and clinical characteristics, lifestyle, physical activity, dietary patterns, genetic and epigenetic profiles and data derived from metabolomics and metagenomics (126).

#### Clarifying the distinction between precision and personalized nutrition

Although “precision nutrition” and “personalized nutrition” are often used interchangeably, they represent distinct concepts that will shape future research priorities. Personalized nutrition typically refers to tailoring dietary advice to individual preferences and behaviors. Precision nutrition, in contrast, is a scientific framework grounded in mechanistic biology, seeking to understand the genetic, metabolic, hormonal, immunological and environmental factors that cause differential responses to foods. Clarifying this distinction is essential to avoid conceptual drift and to ensure that precision nutrition advances as a rigorous, translational science.

#### Emerging tools and large-scale initiatives

Precision nutrition employs a data-driven approach that integrates diagnostic tools, biomarkers and diverse 'omics' technologies—including genomics, metabolomics and metagenomics—to evaluate individual responses to foods (123, 124, 127, 128). Ongoing initiatives use these approaches to identify dietary factors influencing health and disease and to design tailored recommendations based on age, life stage, gender, ethnicity, metabolic status and medical conditions.

In the United States, the NIH's Nutrition for Precision Health initiative—supported through the *All of Us* research program—is investigating interactions among diet, genetics, the microbiome, metabolism and other personal attributes, while developing artificial intelligence (AI) algorithms capable of predicting individual reactions to dietary patterns (125).

The British Personalized Responses to Dietary Composition Trial (PREDICT) series (PREDICT 1, 2 and 3) likewise examines how genetics, gut microbiome composition, metabolic differences, exercise, sleep, time of day and meal composition influence postprandial responses. These studies leverage mobile applications and wearable devices to continuously capture glucose profiles, physical activity and sleep patterns (133–136). The Food4Me randomized controlled trial also demonstrated that individualized nutrition advice—delivered digitally across seven European countries—resulted in greater improvements in dietary behavior than conventional non-personalized guidance (137, 138).

Together, these initiatives underscore that interindividual variability in dietary responses is substantial, predictable and biologically meaningful.

#### Population-level relevance and structural context

Although precision nutrition emphasizes individual biological differences, its goals align with public health. Individuals' dietary choices are influenced not only by personal factors, but also by broader structural elements such as food systems, access, affordability, culture, and policy. Consequently, precision nutrition must address these structural determinants, which influence dietary patterns and may limit the practicality of individualized recommendations. Looking ahead, effective precision nutrition frameworks will need to integrate considerations around food systems and sustainability, ensuring guidance is compatible with environmental and economic realities. They should also address lifecycle-specific nutritional needs, recognizing that different population groups have unique vulnerabilities. In addition, promoting digital and diagnostic equity is essential to avoid exacerbating existing disparities in diet-related diseases as precision nutrition tools advance. In this sense, precision nutrition should not be viewed as a consumer-level customization tool, but rather as a methodology to refine population-level recommendations by revealing where “average” guidance fails and how subgroups differ in predictable, biologically grounded ways.

#### Mechanistic grounding as the next frontier

To fully realize the promise of precision nutrition, the field must deepen its focus on understanding the underlying biological mechanisms that drive individual differences in dietary responses. While large multimodal datasets have revealed the extent of interindividual variation, it remains crucial to clarify the biological reasons behind these differences and their implications for health outcomes. Priority areas for mechanistic discovery include the genetic and epigenetic factors shaping nutrient metabolism and dietary effects; the interplay between the microbiome, immune system, and metabolic processes influencing disease risk and aging; the complex roles of lipoprotein particle biology and vascular function—such as how HDL subclasses impact inflammation and cerebrovascular health; and major neuroendocrine transitions like puberty and menopause that alter nutrient needs and metabolism. Anchoring predictive models within biological pathways will transform precision nutrition from descriptive variability to actionable insight.

#### The role of AI and digital phenotyping

Precision nutrition is rapidly advancing due to AI and wearable sensor technology. AI platforms integrate data from genetics, microbiomes, diet, and real-time metrics to create personalized nutrition plans. Machine-learning models detect complex interactions between diet and health, enabling predictive, individualized recommendations. A review of 60 studies found that ML is essential for integrating complex data in precision nutrition research (139). Wearable devices like glucose monitors and smartwatches now track not only activity but also meals, nutrient intake, and biomarkers, offering continuous data for AI analysis (133, 140, 141). This shift supports a move from general guidelines to adaptive, person-specific interventions and is already being applied in clinical areas such as critical care and chronic disease management (142). Key challenges include data accuracy, sensor reliability, user adherence, bias, privacy, and the need for strong clinical validation. As noted by Sempionatto et al., combining multiple sensor types with advanced analytics is crucial for effective precision nutrition models (143). With ongoing improvements, the integration of AI and wearables is expected to accelerate discoveries and bring data-driven nutrition into everyday healthcare, helping prevent metabolic and chronic diseases.

#### Future priorities (2025–2030)

To support meaningful progress in precision nutrition, research should focus on establishing clear conceptual definitions distinguishing between precision, personalized, and population-level nutrition. It is essential to identify response phenotypes that reflect significant biological differences across various life stages and demographic groups. Integrating mechanistic insights by connecting omics data to specific metabolic, vascular, endocrine, and neurological pathways will deepen understanding of dietary effects. These precision findings should then be translated into population health strategies, enabling more targeted dietary recommendations where appropriate. Ensuring equity and representativeness in data collection is vital so that advances benefit diverse populations. Finally, precision nutrition must incorporate food composition data and address food-system constraints, guaranteeing that recommendations are practical, environmentally sustainable, and applicable to real-world settings.

Precision nutrition represents an important evolution in nutrition science, linking mechanistic biology, multimodal data, population diversity and real-world dietary environments. To fulfill its promise, the field must advance from pattern recognition toward mechanistic understanding, embed biological heterogeneity within a population-health framework and ensure equitable access to precision tools. By addressing these challenges, precision nutrition can enhance resilience and health across diverse human populations and stages of life. As proof of growing research activity in the field, the quantity of scientific publications is rising at a marked pace and is anticipated to increase significantly in the near future. This trend is reflected by the growing number of articles featured in both the Nutrigenomics and Nutrition and Microbes sections of our journal. The number of published papers has risen from less than 15 in 2019 to about 200 projected by 2025, with expectations for continued exponential growth through 2030.

## Gut microbiota – diet interactions: from association to causation

### (Maria Carmen Collado)

Advances in omics technologies (metagenomics, metabolomics, etc.) and their integration with dietary data have highlighted the microbiome-diet-human health interactions pointing out a major role in nutritional status, metabolic regulation, and immune function, influencing human health. However, current research is still limited due inconsistent methodologies, uncovered mechanistic and functional insights, poor standardization, a lack of validated biomarkers, and fragmentation between clinical, agricultural, environmental, and food system perspectives. In addition, most of the scientific evidence relies on small sample sizes, cross-sectional study designs, and short-term impact in limited demographic/geographic/economical contexts. Then, a priority in the field is the implementation of standardized sampling protocols, harmonized dietary metadata frameworks, and interoperable multi-omics repositories that will allow cross-cohort comparisons, reproducible research, and identification and validation of microbiome-related biomarkers and clinically meaningful endpoints. All those things are key for the progress and support of precision nutrition, and also, advances on the regulatory/clinical evaluation and validation. Recent efforts have provided specific reporting microbiome standards such as the STORMS checklist (144) and the STREAMS guidelines (145), as well as the availability of large-scale microbial catalogs, including the food microbiome dataset (146), multi-location microbiome datasets (147, 148) and others (149). In parallel, new guidelines for equitable and collaborative microbiome data reuse of have been proposed, such a scientific community -driven roadmap microbiome data sharing (150). In addition to all these efforts, it is needed to establish robust governance frameworks that address privacy, consent, equitable benefit sharing, and ethical commercialization of microbiome data.

To advance in nutritional science, microbiome research needs to progress from associations and inferences to establishing causality. This requires a deep understanding of how different dietary patterns, specific nutrients, bioactives, and food components shape microbial ecosystems and their metabolic functions with impact on inflammation, metabolic regulation, developmental programming, and health trajectories across the lifespan (151). All these will guide targeted microbiome-modulating strategies, including targeted fibers and prebiotics, probiotics, and other biotics (synbiotics and postbiotics), fermented foods, and live biotherapeutics (152). However, advances in this area depend on well-designed clinical trials conducted in well-defined populations (healthy and/or at risk), with standardized reporting including both mechanistic and clinical outcomes (153). On the other side, integrating microbiome science into nutrition policy and regulation, particularly in food additives, processing methods, safety assessments, and health claims, remains an unmet need. Precision nutrition approaches will be enhanced by linking microbiome data with host genetics, metabolomics, and dietary profiles while ensuring model validity across diverse populations to avoid amplifying health inequities. Moreover, microbiome research has also the potential to address under/over nutrition, growth faltering, metabolic disease, and the development of personalized or culture-tailored foods in both high- and low-resource settings. This aligns with the “One-Health” approach (154), which recognizes soil, plant, animal, and environmental microbiomes as interconnected determinants of nutritional quality, food diversity, and health outcomes. This approach promotes sustainable, safe, and equitable global food systems that optimize nutritional wellbeing, representing one of the main goals for the microbiome-nutrition field.

## Performance nutrition

### (David C. Nieman)

Sport and exercise nutrition is focused on the optimization of dietary strategies to support and enhance training adaptations, performance, health, and metabolic recovery (155–157). Commercial and research interest in sports nutrition has expanded rapidly in the 21st century. The global sports nutrition market was worth $45 billion in 2024 and is projected to reach $86 billion by 2032 (158). The number of academic journal articles focused on sports nutrition has grown exponentially since 2000 (159–161). Based on available data at journal websites, the four leading journals for articles on sports nutrition are *Nutrients* (235 articles in 2024, impact factor, IF, 5.0), *Frontiers in Nutrition* (66 articles in 2024, IF 5.1), *Journal of the International Society of Sports Nutrition* (65 articles in 2024, IF 3.9), and the *International Journal of Sport Nutrition and Exercise Metabolism* (43 articles in 2024, IF 2.6). The two newest journals with sections devoted to sports nutrition (*Nutrients* and *Frontiers in Nutrition*) are open source and during a 5-year period increased the number of articles from 88 in 2020 to 301 in 2024. This growth should accelerate as research methodologies and technologies improve in parallel with the shift toward personalized sports nutrition in academic, commercial, and public sectors (156). Additionally, companies now offer a wide variety of sports nutrition products to everyone from athletes to fitness enthusiasts and are sponsoring more studies in university settings. Physical activity and nutrient intake are monitored by exercisers and researchers across the globe using phones, smart watches, and wearable sensors (156, 160, 162).

Surveys show that a majority of athletes use dietary supplements to support day-to-day training stress and recovery, and competitive performance (163). Popular sports nutrition products include protein products, sports beverages, powders, and foods such as bars and gels. These products are convenient and marketed to physically active people at all performance and age levels. Sports supplements have been examined repeatedly in many studies and those that are efficacious for performance and metabolic recovery include carbohydrates, proteins and amino acid mixtures, caffeine, creatine, β-alanine, nitrate/beetroot juice, sodium bicarbonate, and polyphenols (164–172).

At the same time, there is an increasing emphasis on a food-first approach in sports nutrition that may reduce the market share for processed sports supplements while increasing the demand for sports foods such as bananas, berries, sweet potatoes, rice, lean proteins (chicken, fish, beans, eggs), nuts, and milk (173). A food-first approach in sports nutrition gives preference to whole foods and juices to meet the nutrient needs of an athlete or exercising individual before resorting to supplements. For the athlete, this approach lowers the risk of consuming supplements that may be mislabeled with prohibited substances (163, 174, 175).

A significant current and future trend is the move toward tailored nutrition recommendations based on the athlete's unique genetic, molecular, and metabolic characteristics (156). This system biology approach is based on a sophisticated and high-priced level of science with advanced multiomics technologies, methods, and bioinformatics. Scientific approaches in the discipline of precision sports nutrition include genomics, transcriptomics, proteomics, metabolomics, epigenomics, and microbiomics. These approaches will be increasingly used by sports nutrition investigators to better understand the complex metabolic response of exercise and diet interactions with applications at the individual level (156, 160).

For example, gut-derived metabolites related to increased blueberry intake have been linked to a post-exercise anti-inflammatory response (176, 177). However, the generation of metabolites from the gut after blueberry intake varies widely between individuals and may require a greater blueberry intake in some athletes to achieve the same beneficial response (156, 176). The gut microbiome composition has an influence on post-exercise metabolic recovery but also varies substantially between athletes (178). For example, the high prevalence of one gut bacteria species, *Prevotella copri*, in about one in four endurance athletes was linked significantly to a high level of inflammation following vigorous exercise (179). Athletes identified with a high prevalence of *Prevotella copri* may require adaptations to training regimens and greater adherence to an anti-inflammatory diet to quell post-exercise inflammation and improve metabolic recovery. Multiomics-based studies may also reveal gender-and age specific differences in the response to sports nutrition supplements that will influence dosing strategies and the potential for adverse effects. These studies are needed due to significant sports nutrition research gaps in female athletes, and young and old athletes.

There is an increasing interest in sports nutrition products that promote health and environmental sustainability (180–182). The sports nutrition industry is experiencing a shift toward sustainable plant-based products and minimally processed foods. Research and product development are focusing on plant-based proteins from sources like pea and hemp, as well as beverages with natural ingredients (180–182). Studies confirm that plant-based diets are not detrimental to muscular strength and may offer performance benefits, particularly for aerobic exercise. These benefits from plant-based dietary patterns have been linked to higher intakes of carbohydrates, dietary fibers, polyphenols, and antioxidant-related nutrients that help combat inflammation and oxidative stress and support epigenetic adaptations to rigorous exercise regimens (171).

Taken together, sports nutrition research during the next 5 years will be increasingly focused on strong research designs and methods using omics technologies. These data will improve diet and supplement recommendations for athletes and exercisers at the small group or individual level to support enhanced performance and recovery.

## Nutrition for brain health

### (Andrew Scholey)

The journal section “Nutrition, Psychology and Brain Health” recognizes the bidirectional interaction between mental states and brain health. As well as being influenced by diet and nutrition, stress, depression, anxiety, and cognitive status all affect eating behaviors, food choice, appetite regulation, and metabolic processes. Research over the coming years will address how chronic stress influences food reward processing, the impact of mood disorders on dietary pattern adherence, and the mechanisms by which psychological interventions improve dietary behaviors. Additionally, it is increasing important to integrate treatment approaches addressing both mental health and nutritional status in the context of the socioeconomic environment. This is imperative for understanding emerging themes over the next 5 years and beyond.

### Long COVID

The COVID-19 pandemic has affected an estimated 400 million people worldwide with post-acute sequelae of COVID-19 (PASC) or “long COVID,” creating an urgent new priority for nutrition science. Neurocognitive symptoms remain underspecified but including “brain fog,” fatigue and cognitive problems. Approximately 60% of those with PASC experience cognitive dysfunction and 74% experiencing fatigue years after initial infection (183). Cognitive deficits equivalent to 3–6-point IQ losses persist in many affected individuals, with memory, reasoning, and executive function most severely impacted (184).

A comprehensive scoping review of 50 studies published between 2020 and 2025 identified potential nutritional interventions relevant to long COVID management. These included vitamin D, amino acids, multi-nutrient formulations, and microbiota-targeted therapies (185). It is speculated that vitamin D supplementation, antioxidants including quercetin, and L-arginine combined with vitamin C may attenuate Long COVID symptoms, while gut microbiota composition may be associated with symptom occurrence (186). However, the evidence remains fragmented across diverse study designs and populations, highlighting the need for well-powered, mechanistically informed randomized controlled trials (185).

In the context of COVID, research priorities must include identifying nutritional interventions to restore blood-brain barrier integrity, determining optimal macro- and micronutrient requirements during recovery, developing dietary strategies targeting neuroinflammation, and investigating the gut-brain axis role in persistent cognitive symptoms. Given the potential scale of affected populations and absence of treatments, nutrition research for Long COVID represents both a public health imperative and scientific opportunity to advance understanding of diet-brain interactions in post-viral syndromes.

### Ultra-processed foods

The global dominance of ultra-processed foods (UPFs) in modern diets presents an escalating threat to brain health across the lifespan. Controversy remains over UPF umbrella definitions, nevertheless they now account for over 50% of total energy intake in some developed nations (187). The combination of direct neurotoxic effects of UPFs and their displacement of nutrient-rich foods has the potential to damage brain and mental health. A prospective cohort study found that each 10% increase in relative UPF intake was associated with a 16% higher risk of cognitive impairment and an 8% higher risk of stroke (188). These associations were independent of adherence to Mediterranean, DASH, and MIND diets, suggesting food processing itself confers neurological risk beyond dietary pattern. Recent meta-analyses demonstrate a 25–35% excess risk of all-cause dementia in the highest UPF consumption quintile, with longitudinal data linking high-UPF diets to 5% reductions in hippocampal volume (189).

Mechanistic research must elucidate how UPFs compromise brain health through multiple pathways: additive-rich, fiber-poor formulations foster gut dysbiosis, systemic inflammation, and insulin resistance, all potentiating hippocampal shrinkage and disrupting frontostriatal connectivity. Critical questions include identifying specific food additives with neurotoxic properties; determining critical windows of vulnerability across neurodevelopment; understanding why effects on stroke risk are greater among Black populations; and developing biomarkers for UPF-induced brain damage. Early-life UPF exposure during pregnancy and adolescence may contribute to lasting cognitive deficits and increased mental health disorder susceptibility, with maternal consumption initiating intergenerational cycles of poor outcomes. Urgent research is needed on nutritional interventions to reverse UPF-induced brain changes and policy approaches to reduce UPF availability, particularly in vulnerable populations.

### Glucagon-like peptide-1 receptor agonists

The growth of the use of Glucagon-like peptide-1 receptor agonists (GLP-1RAs) has significant implications for nutrition and brain health, including influencing food choice and eating behaviors, as well as in the context of neuroprotection and cognitive function. Alongside their influence on appetite and satiety, these agents modify food preference and ingestive behaviors in ways which are currently poorly understood (190). It has been suggested that reduced energy intake associated with these drugs may increase the risk of micronutrient deficiency (65) and other potentially concerning nutritional risks such as sarcopenia. The indirect health impact of altered food choices should be clarified in the coming years.

GLP-1RA agents act on central and peripheral GLP-1 receptors, contributing to improved neuronal insulin signaling, decreased neuroinflammation, and enhanced neuronal survival and plasticity—all fundamental processes for memory and learning. In clinical and preclinical studies, GLP-1RAs have shown the capacity to preserve cerebral metabolism and brain connectivity, especially in early neurodegenerative disease and in individuals with type 2 diabetes, who are at elevated risk of cognitive decline (191). Early findings suggest GLP-1RAs may improve cognitive performance and lower dementia risk, though robust evidence of their effects on AD biomarkers such as amyloid-β and tau remains inconclusive, and several key questions remain. The exact impact of GLP-1RAs on neurodegenerative disease pathology is still not fully delineated, nor are their long-term cognitive effects in non-diabetic populations. Further investigation is needed to clarify the optimal timing and patient selection for neuroprotection, along with the molecular mechanisms that drive brain health benefits. Well-powered trials are essential to determine whether GLP-1RAs truly modify the course of neurodegenerative disease and to guide clinical application.

### Combating nutrition misinformation in the digital age

The proliferation of nutrition misinformation through social media platforms represents a critical threat undermining evidence-based dietary guidance and public health. Systematic reviews have linked social media use with depressive and disordered eating, and orthorexia nervosa symptoms (192, 193).

Research priorities include: understanding psychological mechanisms by which misinformation influences dietary behavior; investigating how misinformation processing and acceptance varies by source type (influencers, celebrities, journalists) developing evidence-based counter-messaging strategies that combine credibility with emotional resonance; and evaluating digital literacy interventions. Healthcare professionals and academic organizations must leverage social media to disseminate evidence-based knowledge while public health institutions implement strategies to improve digital literacy.

### Methodological imperatives

Achieving these goals requires methodological innovation. Standardized, validated dietary assessment tools with reduced measurement error are essential. Integration of digital technologies—smartphone apps, wearable sensors, artificial intelligence—can improve dietary monitoring while reducing participant burden. Study designs must incorporate n-of-1 trials, adaptive designs, and pragmatic trials embedded within real-world settings.

Crucially, interdisciplinary collaboration must extend beyond lip service. Nutrition scientists, psychologists, neuroscientists, psychiatrists, gastroenterologists, and implementation scientists must work in integrated teams from study conception through dissemination. Training programs should prepare the next generation with cross-disciplinary competencies.

### Translation and implementation

Scientific discoveries must translate into accessible interventions. This requires engagement with public health practitioners, policymakers, and communities to develop scalable, culturally appropriate programs. Special attention should be directed toward populations experiencing health inequities, ensuring that advances benefit rather than exacerbate disparities. The challenge of nutrition misinformation demands new communication strategies meeting people where they are—on social media platforms—with messages combining scientific rigor with emotional resonance. Public-private partnerships may be necessary to counteract well-funded misinformation campaigns, while regulatory frameworks must address misuse of medical credentials and deceptive marketing practices.

The 2025–2030 period offers great opportunities to transform understanding of the reciprocal relationship between nutrition, psychological wellbeing and brain health. The emergence of Long COVID, recognition of UPF neurotoxicity, widespread adoption of GLP-1 agonists and proliferation of digital misinformation have created urgent imperatives alongside ongoing priorities in mechanistic research and precision nutrition.

## Interdisciplinary systems thinking

### (David Raubenheimer)

“*… if we want to bring about the thoroughgoing restructuring of systems that is necessary to solve the world's gravest problems … the first step is thinking differently”* [Donella Meadows (194)].

As previous sections illustrate, many nutrition challenges—such as obesity, diet-related chronic diseases, undernutrition, and food system sustainability—are inherently complex, involving biological, behavioral, social, economic, and environmental factors. Reflecting this complexity, calls for nutrition science to adopt interdisciplinary systems approaches, and even proposals for a new science of nutrition, have become common in recent years (195). These critiques typically highlight the limitations of narrowly focused (“siloed”) reductionist paradigms which focus on isolating proximal causal relationships, such as physiological responses to individual nutrients, from their broader context.

Reflecting such concerns, interdisciplinary and/or systems-oriented approaches are already used in many areas of nutrition-related research, including public health nutrition (196), precision nutrition (197), obesity research (198), nutritional ecology (199), food systems research (200), and others. However, adoption remains uneven and often under-applied relative to the complexity and interconnectedness of nutrition, sometimes using the terminology of systems thinking without operationalizing it (196).

An important challenge for nutrition science is to strengthen integration across scales and subfields, connecting mechanistic insights from biological research with systems level understanding of food environments, policy, and sustainability, to optimize solutions-focused research. The aim of this section is to draw attention to some key terms, concepts, and distinctions in interdisciplinary systems science, to help facilitate a mindful evolution toward a better integrated nutrition science. Since space permits only a cursory overview of an extensive topic, the focus will be on the key contrasts between “siloed vs. interdisciplinary” and “reductionist vs. systems,” and how these two continua intersect.

### Cross-disciplinary research

Research that addresses challenges at the interface of biological, social, technological, and ecological systems requires collaborations combining expertise from multiple disciplines and insights gained from lived experience and practical knowledge in real-life settings. Several terms are used to describe the nature of collaborative engagement, but these are seldom explicitly defined and often used interchangeably (201). For present purposes, I will use “cross-disciplinary” collaboration as an umbrella term for research that combines expertise from two or more fields (202), as contrasted with “mono-disciplinary” research. Cross-disciplinary research can be distinguished from mono-disciplinary research and classified into sub-categories as follows:

Mono-disciplinary: A single discipline examines a problem using its own theories, methods, and data, with no formal incorporation of other fields. An example from endocrinology would be a clinical trial examining how leptin and insulin signaling affects appetite and energy balance.

Multidisciplinary: Several disciplines contribute in parallel, each retaining its own disciplinary perspectives and methods with little attempt to integrate across disciplines. This would be the case, for example, if a consortium of researchers from different disciplines participated in a clinical feeding trial and behavioral, neurobiological, endocrinological, and cardiovascular data were collected and analyzed separately with little attempt at integration.

Interdisciplinary: Involves integrating knowledge, methods, and perspectives from different disciplines to address a common problem. An interdisciplinary study might, for example, combine expertise from nutrition science, endocrinology, sociology, and economics to understand obesity as a multi-factorial condition.

Transdisciplinary: Integrates academic expertise with community and stakeholder knowledge to co-create solutions for complex real-world problems. An example is the design of a community-based obesity prevention program that combines academic expertise (e.g., nutrition scientists, public health researchers, behavioral scientists) with community knowledge (residents' experiences, local businesses, policymakers). Typically, the scientific evidence would be integrated with community knowledge to co-design actionable interventions.

These terms are most frequently used to describe the nature of research projects, programs, institutes, and occasionally scientific fields, but they can also refer to collaborative relationships within each of these contexts (203). This highlights that a research project, program, or institute may simultaneously include mono-, multi-, inter-, and trans-disciplinary elements. The optimal mix should be determined by the research goals.

Regardless of the configuration of disciplinary relationships, templates are needed to select, combine, and integrate relevant expertise in ways that best fit the research aims. Systems thinking and systems frameworks are powerful approaches for this, as outlined below. It should, however, first be emphasized that systems approaches are not the only integrating tool—others include Design Thinking, Participatory Approaches, and Integration and Implementation Science. Second, like modes of cross-disciplinary engagement, these approaches are not mutually exclusive; they can be combined within a single research program to harness complementary strengths (204).

### Systems thinking

Systems thinking (ST) can be understood as a holistic approach that frames problems as emerging from systems of interacting components, rather than being “caused” by any single factor (205). In a practical context, ST acknowledges that system change or management typically involves trade-offs, because the interconnectedness of systems means that interventions to improve one output might also produce (possibly negative) changes in others. For example, healthy dietary patterns might be less environmentally sustainable or be less affordable (206). This is fundamentally important for complex societal problems, in which stakeholder incentives are often misaligned, and in physiological contexts, where interventions can lead to unintended side effects.

ST is sometimes portrayed as an antidote to reductionist research. This is incorrect—it is a category error that contrasts a way of framing problems (ST) with the level of organization at which the problem is addressed. Rather than replacing reductionism, ST should guide the strategic integration of insights across the reductionist-synthesis spectrum, from physiological interactions with nutrients to the dynamics of food systems (199).

Two points illustrate this. First, ST can include reductionist analysis, for example to understand the properties of the system components. Second, “reductionist” research can itself employ ST, as in metabolic research that applies systems approaches, or precision nutrition (above). This latter point illustrates that systems components are usually themselves systems (termed sub-systems), and thus amenable to the full range of considerations relevant to systems research.

These theoretical considerations can have important practical implications. For example, the problem of ultra-processed foods cannot be solved using only reductionist research that examines how the properties of foods interact with physiology—it is a problem rooted primarily in industry, policy, and consumer behavior. On the other hand, physiological research is important because a lack of evidence on the biological mechanisms remains a significant impediment for policy interventions (207).

Some key considerations in ST include how to delineate the system most relevant to the research challenge, and how to map the interconnections among components. These and other challenges in operationalizing systems thinking are addressed using systems frameworks.

### Systems frameworks

Systems frameworks provide a structured approach to implementing systems thinking, for example in conceptualizing, analyzing, designing, and managing a system. Typically, systems frameworks stipulate a boundary (the scope of factors considered relevant to the research problem), define components (or sub-systems) considered important to the system's functioning, and at varying levels of granularity postulate interactions of system and sub-system components. Many systems frameworks have been developed in nutrition, across scales from intracellular (e.g., mTOR, AMPK, and insulin signaling) to global (e.g., food systems frameworks).

Systems frameworks can be either conceptual or methodological. Conceptual frameworks provide theoretical structure for understanding dynamics within a system. Nutritional ecology is a conceptual framework that depicts nutrition as comprising the organism, the environment, and the nutrition-related interactions of organism and environment (208). Systems frameworks discussed in previous sections include *One Health* and the SDG framework. Methodological systems frameworks operationalize conceptual systems frameworks through guiding the collection, analysis and interpretation of data. A micro-scale example is genome-scale metabolic models, which are computational representations of an organism's entire metabolic network; a macro-scale example is multi-regional input–output analysis (209), which was developed in economics for tracing the flow of goods, services, and associated environmental and social impacts across, for example, food systems.

Systems frameworks are often combined or integrated to address specific problems. For example, Nutritional Geometry (NG), an integrative multi-dimensional framework, was developed in nutritional ecology to model the nutritional interactions of organisms with food environments (208). NG has also been integrated with other systems frameworks. In food systems research, NG and Multi-regional Input–Output analysis (MRIO) were integrated to examine trade-offs between nutritional, environmental, and economic consequences associated with different Australian dietary patterns (206). This is an example of interdisciplinary integration between economics (MRIO) and (nutritional) ecology.

A particularly important class of systems frameworks are complex systems frameworks, in which systems are characterized as composed of many interacting components whose collective behavior has properties that cannot be fully understood by analyzing the components in isolation (210). Two closely related complex systems frameworks are the non-linear systems framework and the complex adaptive systems framework (discussed in a previous section). The non-linear systems framework is mathematically focused, referring to systems where interactions between the components involve feedback loops, thresholds, and non-linear dynamics, and outputs are not proportional to inputs. The CAS framework in the first section has a more conceptual focus, describing systems composed of “agents” that interact, adapt and evolve over time. Many methodological modeling approaches have been developed to implement complex systems frameworks, which are beyond the scope of this brief overview.

Complex systems frameworks are invaluable for tackling problems involving biological, societal, and ecological systems because they capture the dynamic, interconnected, and non-linear nature of these systems. This enables models to represent feedback loops, emergent behaviors, and cross-scale interactions not represented in simpler frameworks. However, this does not mean complex systems frameworks are always the best choice for nutritional research. These models are data-intensive, computationally demanding, and can be difficult to interpret; for many applications, therefore, simpler frameworks are preferable. Depending on the research goal, linear and complex systems frameworks can be combined in models to achieve the best balance of simplicity and realism. For example, Friel et al. (211) combined linear associations—such as between income and diet quality—with non-linear approaches to understand inequities in healthy eating.

Tackling complex challenges require a comprehensive toolkit, from deep disciplinary reductionist research to transdisciplinary systems models. Historically, nutrition science has centered on the mono-disciplinary reductionist ends of the spectrum, but cross-disciplinary systems approaches are increasingly being applied. Their application is, however, patchy and poorly integrated. How can we improve on this? Echoing Donella Meadows' (194) quote above, perhaps the greatest priority is to strengthen the culture of *systems thinking*, empowering nutrition researchers to see their work—regardless of scale or disciplinary breadth—within its broader context. Achieving this will require thoughtful reform in institutional structures (212, 213) and, above all, a reimagining of nutrition education (214, 215), which embeds deep disciplinary training within systems thinking.
